# Human retinal ganglion cell neurons generated by synchronous BMP inhibition and transcription factor mediated reprogramming

**DOI:** 10.1038/s41536-023-00327-x

**Published:** 2023-09-29

**Authors:** Devansh Agarwal, Nicholas Dash, Kevin W. Mazo, Manan Chopra, Maria P. Avila, Amit Patel, Ryan M. Wong, Cairang Jia, Hope Do, Jie Cheng, Colette Chiang, Shawna L. Jurlina, Mona Roshan, Michael W. Perry, Jong M. Rho, Risa Broyer, Cassidy D. Lee, Robert N. Weinreb, Cezar Gavrilovici, Nicholas W. Oesch, Derek S. Welsbie, Karl J. Wahlin

**Affiliations:** 1 Shu Chien-Gene Lay Department of Bioengineering, UC San Diego, La Jolla, CA USA; 2 Viterbi Family Department of Ophthalmology & the Shiley Eye Institute, UC San Diego, La Jolla, CA USA; 3 Department of Ophthalmology, Johns Hopkins School of Medicine, Baltimore, MD USA; 4 Department of Biological Sciences, UC San Diego, La Jolla, CA USA; 5 Department of Neurosciences, UC San Diego, La Jolla, CA USA; 6 Department of Psychology, UC San Diego, La Jolla, CA USA

**Keywords:** Reprogramming, Regeneration

## Abstract

In optic neuropathies, including glaucoma, retinal ganglion cells (RGCs) die. Cell transplantation and endogenous regeneration offer strategies for retinal repair, however, developmental programs required for this to succeed are incompletely understood. To address this, we explored cellular reprogramming with transcription factor (TF) regulators of RGC development which were integrated into human pluripotent stem cells (PSCs) as inducible gene cassettes. When the pioneer factor *NEUROG2* was combined with RGC-expressed TFs (*ATOH7*, *ISL1,* and *POU4F2*) some conversion was observed and when pre-patterned by BMP inhibition, RGC-like induced neurons (RGC-iNs) were generated with high efficiency in just under a week. These exhibited transcriptional profiles that were reminiscent of RGCs and exhibited electrophysiological properties, including AMPA-mediated synaptic transmission. Additionally, we demonstrated that small molecule inhibitors of DLK/LZK and GCK-IV can block neuronal death in two pharmacological axon injury models. Combining developmental patterning with RGC-specific TFs thus provided valuable insight into strategies for cell replacement and neuroprotection.

## Introduction

Glaucoma, a leading cause of irreversible blindness, and other optic neuropathies are characterized by retinal ganglion cell (RGC) death secondary to axonal injury. Cell transplantation and endogenous regeneration offer the possibility of restoring RGCs, however, the developmental programs required for differentiation and maturation are not completely understood. In several vertebrate species, including zebrafish, a remarkable regenerative capability exists, but in mammals it is limited. Presumably, endogenous regeneration of RGCs requires precise control over the composition and timing of developmental transcription factors (TFs)^1^. Improved knowledge of these could lead to new approaches for generating transplant-ready RGCs, endogenous repair and a platform for rapidly assessing neuroprotective therapeutics.

The developing central nervous system (CNS) is comprised of a diverse collection of cells that form through the combined action of TFs. Early in development, morphogens regulate Lhx2, Pax6, Rax, Six3, and Six6 which in turn coordinate eye and forebrain development. Bone Morphogenetic Protein 4 (Bmp4), which is necessary for retina specification^2^, activates Sox2, while Bmp7 activates Pax6^3^. BMP4 also influences dorsal patterning^4^ while Sonic hedgehog (Shh) cooperates with SIX3 to specify ventral forebrain and neural retina. At later stages, Neurogenin-2 (NEUROG2), Atonal homolog-7 (ATOH7), Islet-1 (ISL1), and Achaete-scute homolog-1 (ASCL1) regulate cell-cycle exit, RGC development, differentiation and cell survival^5,6^. NEUROG2 alone promotes neurogenesis, cell specification, differentiation and migration in vivo^7,8^ and in human pluripotent stem cells (PSCs) it can trigger neural conversion^9–11^. In chickens and mice, it coordinates with Atoh7 to generate immature RGCs from cultured retinal pigment epithelial (RPE) cells^5^. Downstream, Isl1 and Pou4f2 (Brn3b) participate in RGC differentiation^12,13^. Their loss delays axon elongation and leads to axon pathfinding errors^13^, optic nerve hypoplasia and cell death^14,15^. In mice, Atoh7 and Pou4f2 can also facilitate partial Müller to RGC reprogramming^16^. Last, the Sry-related high mobility box (Sox) superfamily SoxC subfamily (Sox4, -11, and -12) influences directional axonal growth and the formation of contralateral RGC axons^17^. They also antagonize Hes5, a suppressor of RGC differentiation; its loss results in a complete absence of RGCs^18^.

Methods for generating human PSC-derived RGCs already exist, yet they are slow and require immunopanning to attain high purity^19^. TFs alone or in combination with small molecule chemicals can drive neural specification and fine-tuning of these combinations may lead to enriched populations of RGCs in less time^10,20,21^. To explore this type of combination in RGCs, we engineered human PSCs with doxycycline (dox) inducible *NEUROG2*, *ATOH7*, *ISL1*, and *POU4F2* transgene cassettes integrated individually or in combination into a single safe harbor site (SHS). NEUROG2 promotes general neurogenesis while ATOH7, ISL1, and POU4F2 promote RGC differentiation. We also discovered that we could significantly enhance TF-mediated conversion by blocking BMP signaling with the small molecule LDN-193189 (LDN). When combined with TFs, LDN facilitated the rapid and efficient formation of RGC-like induced neurons (RGC-iNs) with long, branched neurites and POU4F2-tdTomato reporter expression in up to 94% of cells. Transcriptional profiles of RGC-iNs across 3 genetic backgrounds, as well as electrophysiological studies, were consistent with an RGC-like identity. Single-cell RNA-seq (scRNA-seq) further corroborated the identity of RGC-iNs and shed additional light on RGC subtype diversity. Last, we demonstrated that RGC-iNs were susceptible to pharmacological axon injury which could be blocked by neuroprotective compounds inhibiting the dual leucine zipper kinase (DLK, MAP3K12) and related leucine zipper kinase (LZK, MAP3K13) signaling pathways. Together, these findings demonstrated that TF reprogramming and chemical patterning synergized to enhance RGC-like neuron formation, making these a practical alternative for studies of RGC development and neuroprotection.

## Results

### Combined overexpression of NEUROG2, ATOH7, ISL1, and POU4F2 leads to the direct conversion of RGC-iNs

**Fig. 1:**
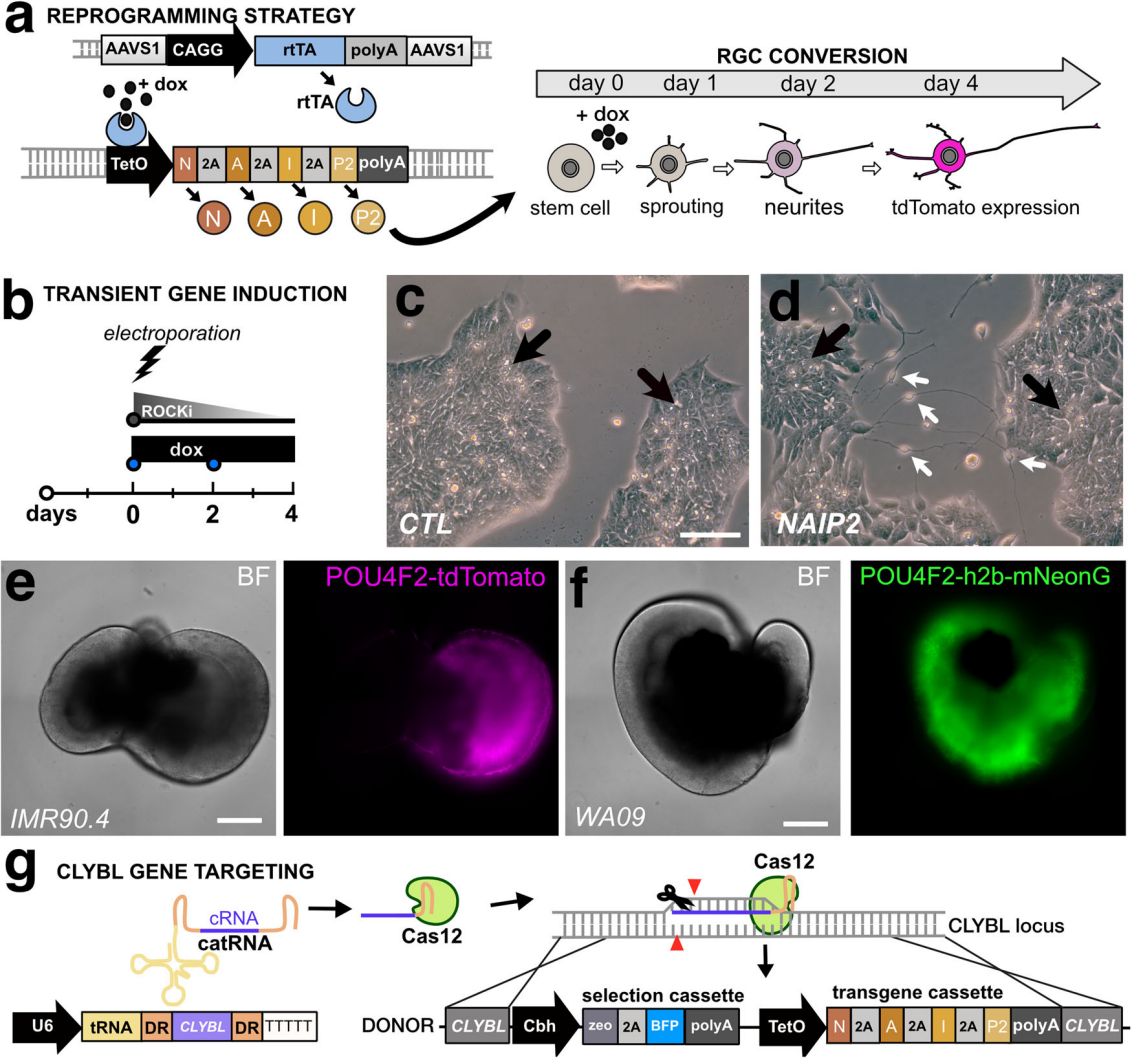
Vector design for insertion of a Tet-On regulated RGC cassette. **a** A conceptual diagram for a 3G Tet-ON system where doxycycline binds to constitutively expressed rtTA to induce polycistronic expression of *NEUROG2*, *ATOH7*, *ISLET1*, and *POU4F2* (*NAIP2*) leading to conversion of RGC-like neurons. **b** Electroporation approach for the transient transfection of the NAIP2 transgene package. **c** PSCs that were transiently transfected with control (empty) or **d** *NAIP2* plasmids and treated with dox for 3 days. Black arrows = PSCs, white arrows = converted neurons. Brightfield and fluorescent images of **e** the POU4F2-tdTomato+ reporter in a 66-day-old retinal organoid in the IMR90.4 genetic background and **f** a 65-day-old POU4F2-h2b-mNeonGreen+ retinal organoid in the WA09 genetic background. **g** U6 promoter-driven expression of an AsCas12a cRNA targeting the CLYBL safe harbor site for insertion of a zeocin selectable Tet-inducible transgene cassette. Scale **c**, **d** = 100 µm, **e**, **f** = 400 µm.

NEUROG2, ATOH7, ISL1, and POU4F2 participate in RGC development and maturation. To explore the idea that their forced expression could convert stem cells directly into RGCs, we used a doxycycline (dox)-inducible third-generation Tet-ON (TetO) promoter coupled to a polycistronic *NEUROG2*, *ATOH7*, *ISL1*, and *POU4F2* (NAIP2) gene cassette (Fig. 1a). After transient transfection into IMR90.4 POU4F2-p2A-tdTomato cells (Fig. 1b) we observed that untreated controls (CTLs) had a PSC-like morphology (Fig. 1c) and dox treated TetO-*NAIP2* transfected cells were PSC-like but also included sparsely populated neurons (Fig. 1d). These were not POU4F2-tdTomato+ and the neuronal looking cells were quickly overtaken by proliferating PSCs. To confirm that the reporters were functioning properly, the IMR90.4 POU4F2-p2A-tdTomato and WA09 POU4F2-p2A-h2b-mNeonGreen PSC lines integrated with TetO-*NAIP2* were differentiated as 3D retinal organoids and confirmed to express fluorescent proteins only in RGCs (Fig. 1e, f). Encouraged by the partial morphological conversion of stem cells into RGCs, we next stably integrated the TetO-*NAIP2* cassette into the *CLYBL* SHS, a site that supports sustained transgene expression^22^. Integration was accomplished with high-fidelity EnCas12a and an appropriate cRNA for gene targeting (Fig. 1g). Despite stable integration, as determined by the acquisition of zeocin resistance, dividing non-neuronal flat cells remained problematic for the long-term growth of the induced neurons.

### BMP inhibition and TF overexpression synergistically enhance the morphological conversion of iNs

**Fig. 2:**
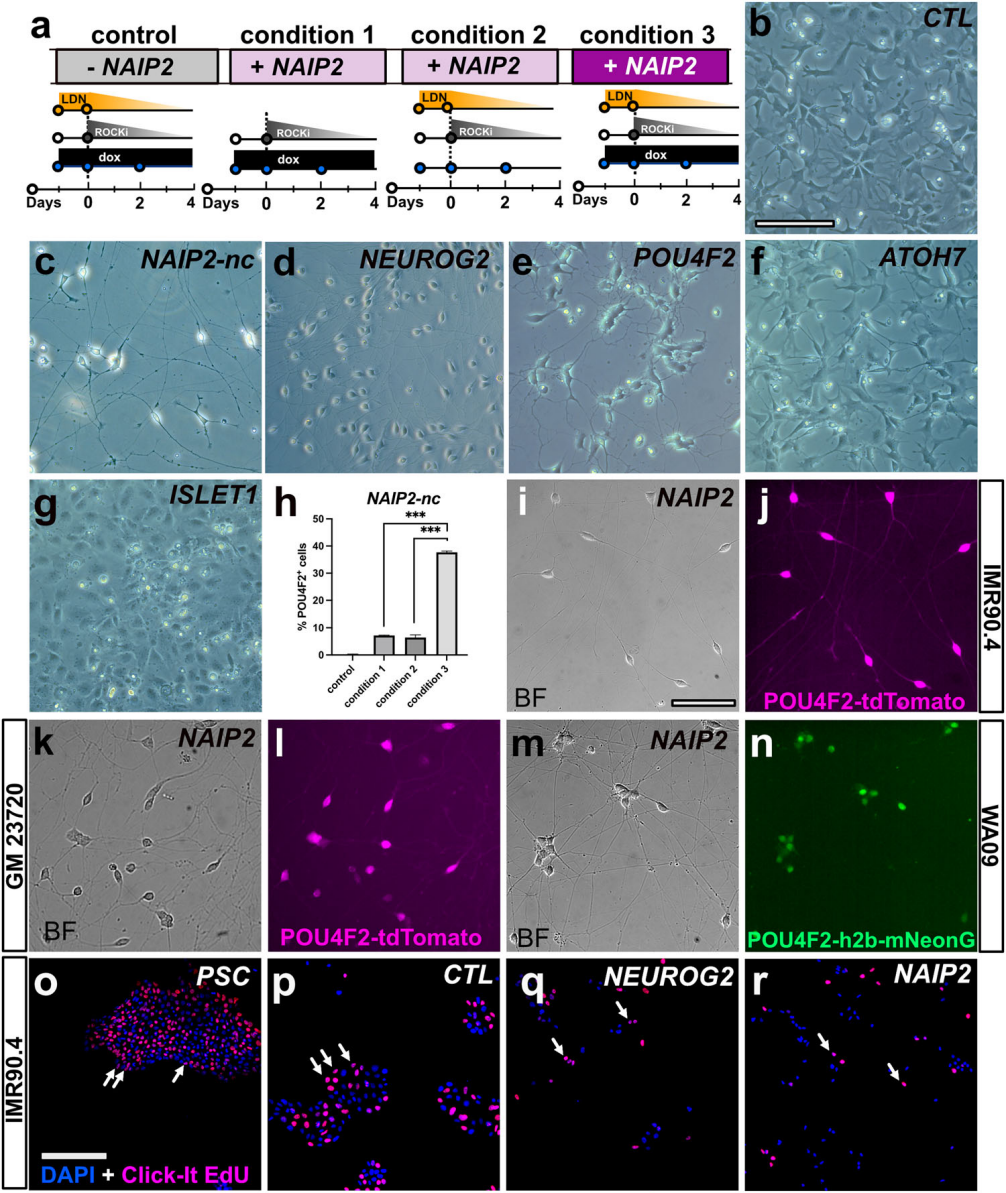
Enhancement of neuronal morphology after patterning by BMP inhibition. **a** Timeline of LDN193189 and/or dox treatments of POU4F2-tdTomato PSCs with an integrated empty cassette (control) or NAIP2 cassette (conditions 1–3). Brightfield images of dox treated **b** empty cassette control cells, **c** NAIP2-nc, **d** NEUROG2, **e** POU4F2, **f** ATOH7, and **g** ISLET1 cells after 6 days. NAIP2-nc = not clonally selected. **h** Quantification of %POU4F2+ cells at day 6 relative to DAPI in the empty cassette control and conditions 1–3 illustrated in (**a**). *P < 0.05, **P < 0.01, ***P < 0.001, ns *P* > 0.05, *n* = 3. Error bars are reported as standard deviation (SD). **i**–**n** *NAIP2* RGC-iNs from different genetic backgrounds differentiated and imaged in brightfield and POU4F2+ tdTomato fluorescence for clonally selected **i**, **j** IMR90.4 and **k**, **l** GM23720 PSCs, and **m**, **n** brightfield and POU4F2 + h2b-mNeonGreen fluorescence for WA09 PSCs. Click-iT EdU staining in **o** undifferentiated PSCs and 2-day LDN/dox treated **p** control, **q** NEUROG2, and **r** NAIP2 cells co-stained with DAPI. Arrows in O-R indicate EdU+ cells. Scale **b**–**g**, **i**–**n** = 100 µm, **o**–**r** = 200 µm.

Simultaneous expression of *NEUROG2* and dual SMAD inhibition (to block BMP and TGF-β) can generate human patterned forebrain-like induced neurons (hpiNs)^10^. To explore whether this could be adapted for RGCs, we simultaneously expressed *NAIP2* and inhibited BMP signaling. IMR90.4 POU4F2-tdTomato reporter PSCs harboring empty (CTL) or *NAIP2* cassettes were treated with 1.0 μg/mL dox (condition 1), 100 nM LDN (condition 2), or LDN plus dox (condition 3) and assessed for changes in morphology (Fig. 2a). Empty TetO cassette integrated CTL cells treated with LDN plus dox failed to generate neurons (Fig. 2b), whereas TetO-*NAIP2* integrated cells (condition 3) developed long branched neurites by day 6 (Fig. 2c). Interestingly, while dual SMAD inhibition with both LDN and SB-431542 (SB) was reported to produce patterned forebrain neurons, its combined use did not improve RGC-iN differentiation more than LDN alone (Supplementary Fig. 1). Although the *NAIP2* combination worked well with BMP inhibition, we wanted to evaluate the contribution of each TF so we integrated each gene (*NEUROG2*, *ATOH7*, *ISLET1*, or *POU4F2*) individually as Tet-inducible cassettes and compared their morphologies after LDN/dox induction (Fig. 2d–g). *NEUROG2* expression led to efficient neural conversion over the first 2 days with long neurites by day 6 (Fig. 2d), whereas neurite formation in NAIP2-nc (NAIP2 integrated and zeocin selected but not clonally isolated) RGC-iNs typically occurred in as little as 24 h, indicating a faster rate of differentiation than *NEUROG2* alone. *POU4F2* overexpressed cells (Fig. 2e) formed stout-looking neurons with stubby neurites that appeared only partially converted, *ATOH7* overexpressed cells (Fig. 2f) became a mixture of flat cells and sparsely populated neurons and *ISL1* cells (Fig. 2g) became flat with no recognizable neuronal features. Overall, only *NAIP2* and *NEUROG2* expressing cells produced robust levels of neurons.

### Endogenous POU4F2 reporter expression accompanies RGC conversion

To corroborate the identity of RGCs, we utilized the POU4F2*-*tdTomato reporter to visualize real-time changes in endogenous POU4F2. Control and NAIP2 PSCs were treated with dox (condition 1), LDN (condition 2), or both (condition 3) and changes in tdTomato expression were evaluated by microscopy. After 1 week, control cells failed to express tdTomato, NAIP2 conditions 1 and 2 had low levels of tdTomato+ and NAIP2 condition 3, which received both LDN and dox, had the highest levels (~40%) of tdTomato+ cells with respect to total DAPI + cells (Fig. 2h).

During RGC-iN formation many flat cells persisted after an initial burst of neurogenesis. We reasoned that cell heterogeneity was limiting conversion so we clonally selected PSCs to obtain PSC lines with greater differentiation potential. Each PSC clone was independently differentiated and the resulting morphologies were observed to range from enriched neurons (Supplementary Fig. 2a, d, e) to unwanted flat cells (Supplementary Fig. 2b, c, f). For all subsequent experiments, a PSC clone with a high conversion efficiency (NAIP2; Fig. 2a) was used. To ensure that conversion was not cell-line dependent, we introduced TetO-*NAIP2* into two additional PSC lines for a total of 3 genetic backgrounds. GM23720 iPSCs and WA09 ESCs were made with POU4F2-p2A-tdTomato and POU4F2-p2A-h2b-mNeonGreen reporters, respectively. In all NAIP2 lines, LDN/dox induced efficient neural conversion (Fig. 2i–n). Further supporting the idea that NAIP2 was driving differentiation, Click-iT EdU (5-ethynyl-2’-deoxyuridine) staining showed a reduced level of cell cycling beyond what was seen in NEUROG2 controls (Fig. 2o–r). Overall, this confirmed that the RGC-iN conversion triggered by *NAIP2* led to early cell-cycle exit and differentiation and was possible across genetic backgrounds.

### Individual transcription factors induce partial reprogramming

**Fig. 3:**
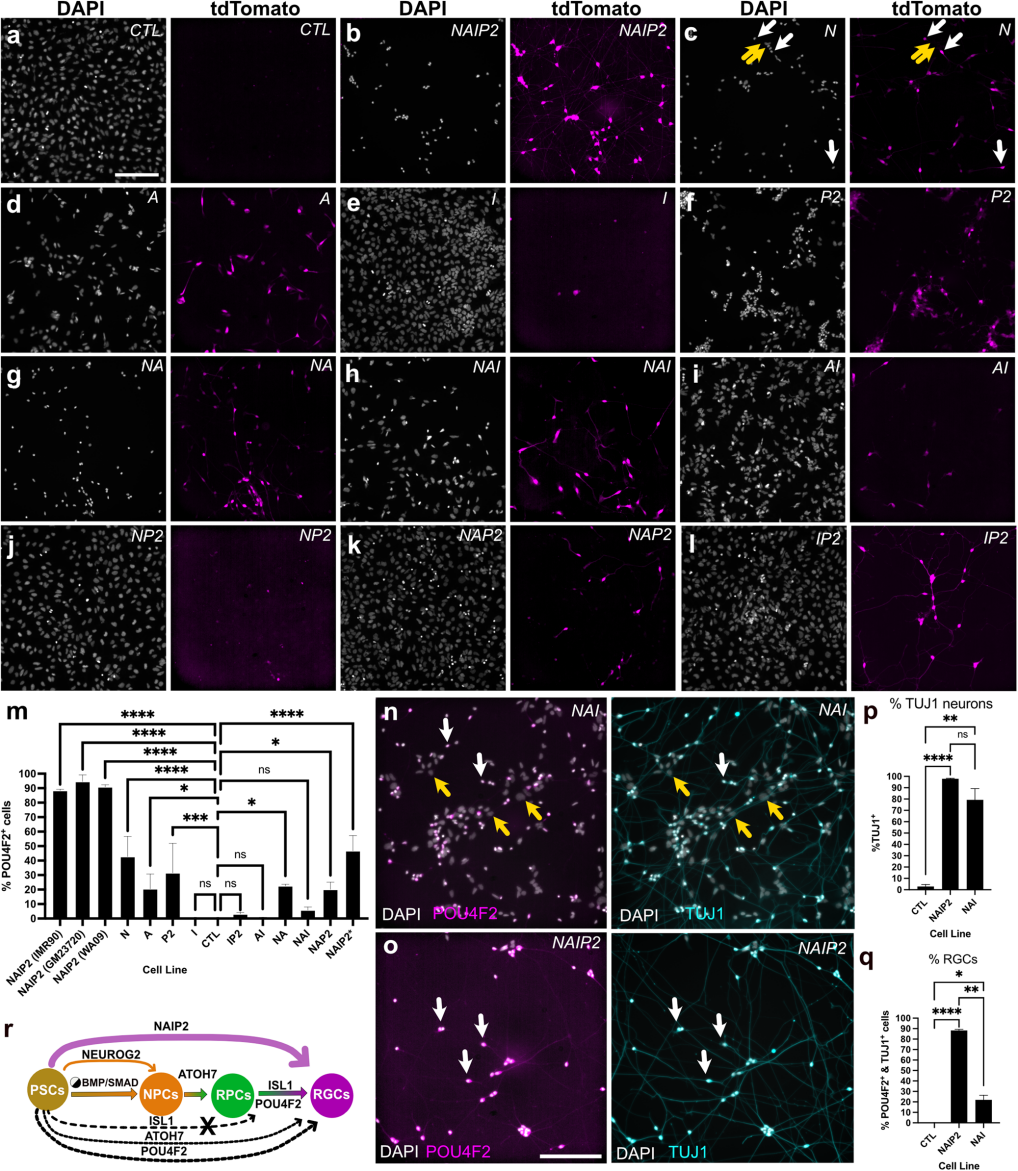
Quantification of POU4F2-tdTomato+ cells induced with different TF combinations. **a**–**l** Representative images of PSCs induced for 6 days with LDN/dox and different TFs alone (N, A, I, P2) or in combination (NAIP2, NA, NAI, AI, NP, NAP2, IP2) with imaging for DAPI (left) and POU4F2-tdTomato+ (right). TdTomato panels are intentionally uniformly overexposed so that samples with weaker fluorescence (**d**–**l**) could be detected. **m** Percent of POU4F2-tdTomato+ cells on day 6 quantified as POU4F2+ cells relative to DAPI. For the WA09 background, this was determined by measuring h2b-mNeonGreen+ relative to DAPI. **n** NAI iNs and **o** NAIP2 RGC-iNs showing POU4F2-tdTomato expression (left) and TUJ1 staining (right). NAI cells were additionally labeled with DAPI. **p** %TUJ1+ neurons and **q** %TUJ1+/POU4F2+ co-labeled RGC-iNs. **r** A diagram summarizing the key events driving differentiation from immature PSCs to mature RGCs. White arrows=overlapping tdTomato+ signal; yellow arrows=absence of a signal. *P < 0.05, **P < 0.01, ***P < 0.001, ****P < 0.0001, ns *P* > 0.05, *n* = 3. Error bars are reported as standard deviation (SD). Scale **a**–**l** = 200 µm; **n**, **o** =100 µm.

We also sought to explore how TFs alone or in combination influenced POU4F2-tdTomato reporter expression (Fig. 3a–l). Compared with controls that showed no fluorescence (Fig. 3a), NAIP2 cells showed high levels of POU4F2-tdTomato+ (Fig. 3b). *NEUROG2* overexpressing neurons exhibited no POU4F2-tdTomato fluorescence at four days, however, a small population of POU4F2-tdTomato+ cells emerged by 6 days (Fig. 3c; white arrows). This was not surprising since POU4F2 is also present in the developing forebrain and *NEUROG2* induces forebrain-like conversion^10^. Though *ATOH7* led to far fewer neurons, those that formed were POU4F2-tdTomato+ (Fig. 3d). Flat *ISL1* overexpressing cells were largely POU4F2-tdTomato negative (Fig. 3e) and *POU4F2* overexpressing cells were only weakly fluorescent (Fig. 3f). This data suggested that while *NEUROG2*, *ATOH7,* and *POU4F2* each contributed, their combined activity was far more effective in triggering neurite outgrowth and endogenous POU4F2-tdTomato gene expression.

Next, we evaluated how neural conversion was influenced by different binary and ternary TF combinations including *NEUROG2*-*ATOH7* (*NA*), *NEUROG2*-*ATOH7*-*ISL1* (*NAI*), *ATOH7*-*ISL1* (*AI*), *NEUROG2*-*POU4F2* (*NP2*), *NEUROG2*-*ATOH7*-*POU4F2* (*NAP2*), and *ISL1*-*POU4F2* (*IP2*) (Fig. 3g–l). In terms of morphology and POU4F2-tdTomato expression, NAIP2 was superior to all binary and ternary TF combinations. The next best was NA (Fig. 3g) which had more POU4F2-tdTomato+ neurons than NEUROG2 but less than NAIP2. NAI (Fig. 3h) produced fewer converted cells and lower levels of tdTomato than NA. Experiments performed with *AI*, *NP2 NAP2* and *IP2* overexpression (Fig. 3i, j, k, l), resembled ISL1 in that most cells were flat and POU4F2-tdTomato negative. Comparisons between different TF combinations (Fig. 3m) also demonstrated that NAIP2 was the most efficient, with conversion efficiencies of 88%, 94%, and 90% in IMR90.4, GM23720, and WA09 PSCs, respectively. While both NAI and NAIP2 generated TUJ1+ neurons (Fig. 3n, o) at efficiencies of 98% and 79%, respectively (Fig. 3p), the number of POU4F2-tdTomato+ neurons was significantly higher for NAIP2 (88%) than for NAI (21%) (Fig. 3q). This demonstrated that while NAI (and other TF combinations) are efficient in making neurons, NAIP2 is the most efficient in making POU4F2 + RGC-like cells. A working model of RGC conversion highlights the cumulative importance of NAIP2 TFs for RGC conversion (Fig. 3r).

### NAIP2 induced RGC-iNs exhibit RGC-like transcriptional profiles

**Fig. 4:**
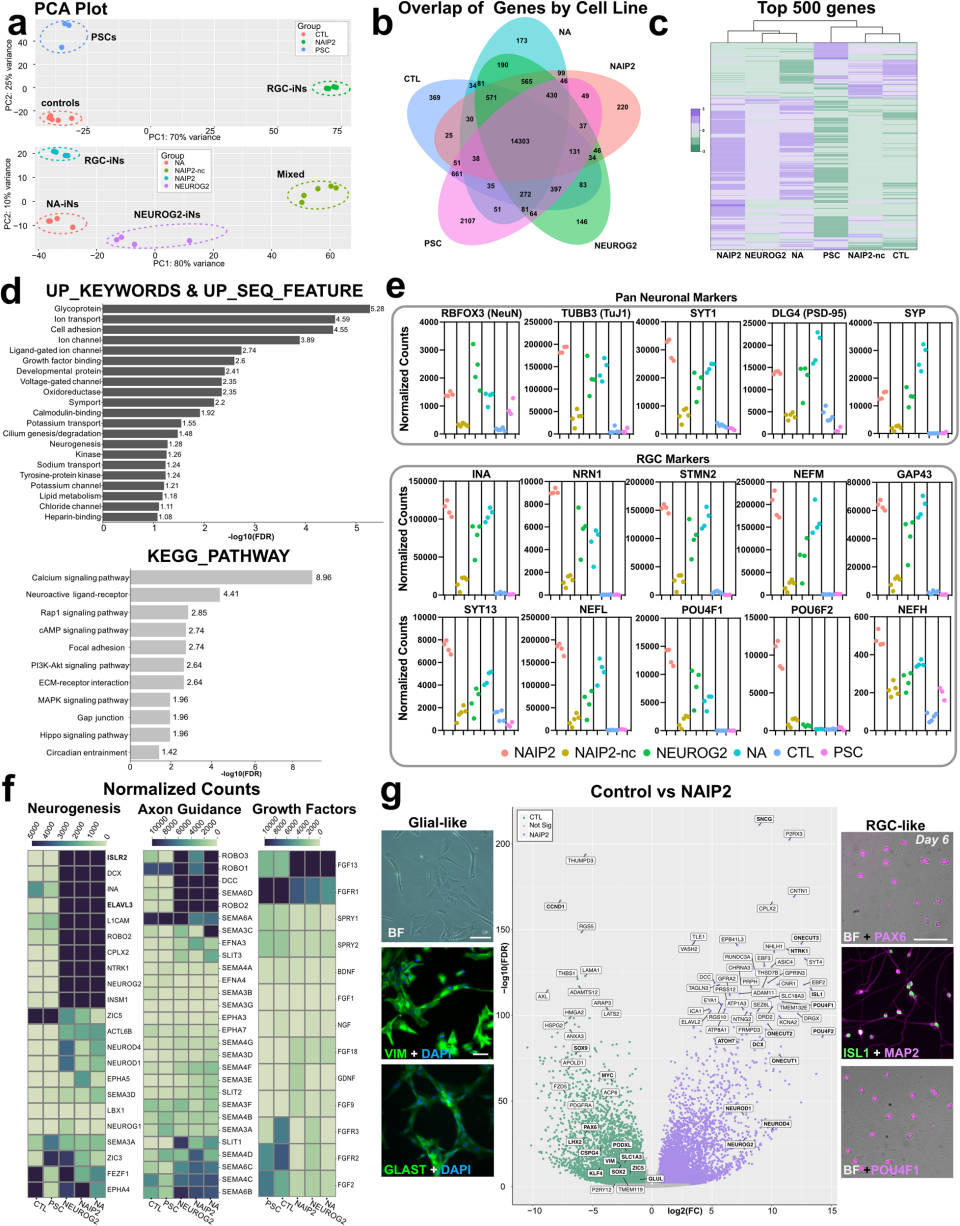
Validation of RGC-iNs by RNA-seq. **a** Principal Component Analysis (PCA) plot of day 6 CTL, NAIP2 and PSC (top) and NEUROG2, NA, NAIP2-nc and NAIP2 cells (bottom). **b** Venn diagram of the number of genes expressed in each sample (normalized count >10). **c** Heatmap of the top 500 genes expressed in the NAIP2 samples. **d** Bar graph depicting −log10(FDR) of significantly differentially expressed pathways between NAIP2 and CTL samples identified by DAVID in Uniprot (top) and KEGG Gene Ontology (bottom) databases. **e** Scatterplots showing normalized counts of pan-neuronal and RGC marker genes in each treatment group. **f** Heatmaps of genes expressed within the neurogenesis (left), axon guidance (center), and growth factor (right) pathways. **g** Volcano plot highlighting log2(FC) gene expression between the CTL and NAIP2 samples plotted with respect to −log10(FDR). Immunostaining for VIM and GLAST in control cells (left) and PAX6, ISL1, POU4F1, and MAP2 in NAIP2 samples (right). FDR = false discovery rate. FC = fold change. Scale **g** = 85 µm. NAIP2-nc = RGC-iNs from non-clonally selected PSCs.

To interrogate the transcriptional identities of induced neurons we carried out RNA-seq on PSCs (*n* = 3), dox-treated CTL (*n* = 5), and *NEUROG2*, *NA,* and *NAIP2* expressing cells (*n* = 4 each) at 1 week (Supplementary Table 1). Principal component analysis (PCA) from DESeq2 demonstrated that biological replicates were consistent across experiments and clustered separately for PSCs, controls and RGC-iNs (Fig. 4a-upper panel). NEUROG2, NA, NAIP2, and NAIP2-nc neurons were also clearly distinct from one another confirming that they had different gene expression profiles (Fig. 4a-lower panel). Global population level differences were illustrated with a Venn diagram depicting the number of genes expressed in each group (normalized count >100) (Fig. 4b). While there was much overlap, treatment groups were distinct from one another. Visualization of the top 500 genes in NAIP2 RGC-iNs (Fig. 4c) revealed that NEUROG2, NA, and NAIP2 groups were more related than controls or PSCs. Zeocin-treated non-clonally selected NAIP2-nc cells consisted of flat cells and neurons while zeocin-treated and clonally selected NAIP2 cells were highly enriched for neurons. The gene expression differences observed in Fig. 4c were consistent with these morphological characteristics. Recognizing the importance of clonal selection, all further analysis was conducted with clonal-selected NAIP2 cells. The top 50 highly differentially expressed genes (DEGs) in NAIP2 cells, including the 4 individual *NAIP2* TFs, were further compared with publicly available single-cell sequencing data which showed a strong similarity to human RGCs as opposed to other retinal neurons (Supplementary Fig. 3a–f)^23^. We also used DAVID pathway analysis (Fig. 4d) to identify biologically relevant signaling pathways in NAIP2 cells relative to control cells. UP_KEYWORDS and UP_SEQ_FEATUREs identified ligand-gated ion channels, voltage-gated ion channels, neurogenesis and potassium/chloride channels, which are all important for neural cell identity and function. Likewise, KEGG_PATHWAYS identified calcium signaling, neuroactive ligand interactions and cAMP/MAPK signaling. In all neurons (NAIP2, NA and NEUROG2) pan-neuronal markers (e.g., *RBFOX3, TUBB3, SYT1, DLG4*, and *SYP*) were detected (Fig. 4e). By contrast, NAIP2 samples expressed higher levels of RGC enriched genes (e.g., *POU4F1*, *POU6F2, NRN1, SYT13*, and *NEFL*), thus supporting their RGC identity. Likewise, we performed RNA-seq on WA09 and GM23720 genetic background NAIP2 cells which similarly expressed RGC genes (Supplementary Fig. 4).

During early RGC growth, there is an active process of axon outgrowth. Both *ISLR2* and *ELAVL3* (Fig. 4f), which are involved in RGC axonogenesis, were expressed in NAIP2 cells but not in PSCs or controls^24^. Functional guidance cues also steer axons towards/away from cellular targets. While some ephrin ligands involved in cell-to-cell communication were highly expressed in non-neuronal controls (e.g., *EFNB1*, *EFNB2*, *EPHA2*) many were upregulated in neurons (e.g., *EFNA3*, *EFNB3*, *EPHA5*, and *EPHB2)*, and in RGC-iNs their expression increased from 1 to 4 weeks (Supplementary Fig. 5a, b). ROBO2/3 and DCC receptors, which mediate RGC axonogenesis, were present in neurons at 1 week (Supplementary Fig. 5c, d)^25^. While *ROBO2/3* decreased over time in RGC-iNs, their ligands *SLIT1* and *-2* increased. Semaphorins (Semas) have different functional roles depending on the tissue type. Plexin (PLXN) family Sema receptors (*PLXNA1*/*3*/*4* and *PLXNB1*) were highly upregulated in RGC-iNs over time (Supplementary Fig. 5e) which makes sense given their role in RGC decussation at the optic chiasm. Although *SEMA3C* was highest in NA cells, it was also detected in other cells including NAIP2 RGC-iNs (Supplementary Fig. 5f). Class 4 (*SEMA4C/D/F*) and class 6 (*SEMA6B/C/D*) Semas, were each temporally upregulated in RGC-, NEUROG2-, and NA-iNs (Supplementary Fig. 5g, h). Conversely, *SEMA6A* was only highly expressed in PSCs and controls, but not neurons, consistent with previous reports^26,27^. Together, these data confirm that RGC-iNs express a rich mixture of axon guidance cues representative of RGC development.

### Blocking BMP signaling without NAIP2 promotes transient glia-like cell formation

Dual SMAD inhibition can drive both neuronal^28^ and glial differentiation^29,30^. While LDN/dox treatment in NEUROG2 and NAIP2 cells led to efficient neural conversion, the same treatment in control cells led to non-neuronal flat cells. A volcano plot highlighted differences between control and NAIP2 cells at 1 week (Fig. 4g). Control samples expressed numerous glia-associated genes (e.g., *VIM*, *SLC1A3* (*GLAST*), and *SOX2*), of which VIM and GLAST were further confirmed by immunocytochemistry (ICC) (Fig. 4g; left). Other glia-expressed genes, including *LHX2* and *SOX9*^31^, were also identified as significantly upregulated relative to RGC-iNs (Fig. 4g). Genes associated with glial proliferation included *CCND1*, *KLF4*, *MYC*, *PODXL*, and *SOX2*, however, these are not exclusive to glia and might indicate immaturity since other glial genes (e.g., *GFAP*, *RLBP1*, and *S100B*) were not detected. Pan-neuronal (e.g., *SYN1*, *NCAN*, *NCAM1, RBFOX3*) and RGC-enriched genes (e.g., *POU4F1, ISL1, GAP43, STMN2,* and *SNCG*) were not expressed in CTLs (Supplementary Fig. 6a). PAX6, ISL1 and POU4F1 were additionally confirmed to be expressed in neurons by ICC (Fig. 4g, right). *PAX6*, which is known to be expressed in both neurons and glia, was also detected in differentiated CTL cells. While CTLs initially resembled glia, subsequent analyses up to 3 weeks revealed that glial markers became reduced (e.g., *VIM*) or disappeared altogether (e.g., *GLAST*) (Supplementary Fig. 6a). While we still do not know the cellular identity of these cells, gene set enrichment analysis (GSEA) revealed epithelial-to-mesenchymal transition (EMT) as an important active process (Supplementary Fig. 6b, c). Some EMT-associated genes, including *ALX4*, *FOXC2*, *GSC*, *MSX1/2*, *NR2F1*, *SNAI2 (SLUG)*, *SNAI3 (SMUC)*, *TGFB1/2*, and *TWIST1* were readily detected, however others, including *CCL5*, *FOXC1*, *SNAI1 (SNAIL)*, and *ZEB1/2*, showed very little expression (Fig. 6d, e).

### Temporal regulation of gene expression reflects neuronal maturation

**Fig. 5:**
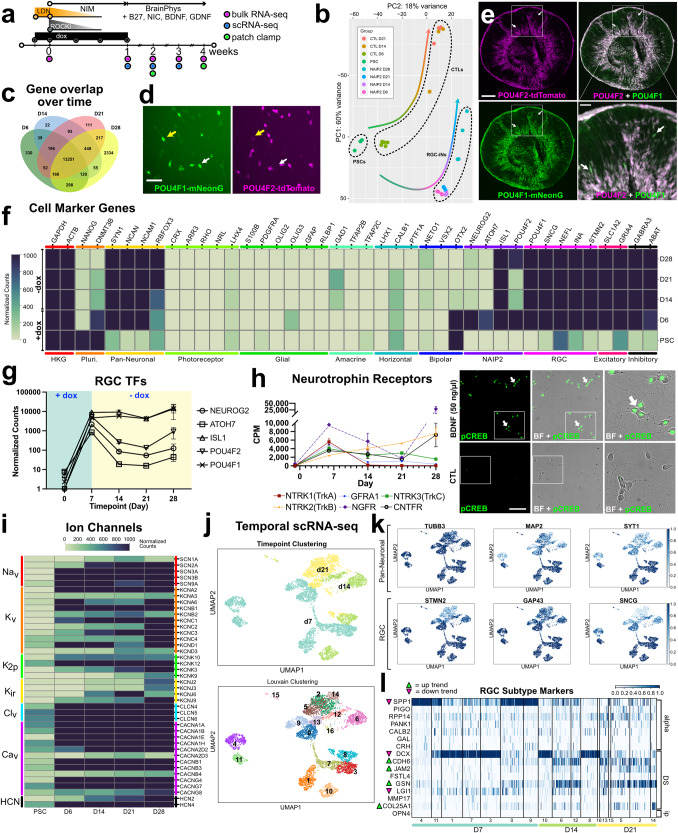
Temporal RNA-seq of RGC-iNs. **a** Timeline for longitudinal studies. **b** RNA-seq PCA plot showing clustering among PSCs, days 6, 14, 21, and 28 (D6, -14, -21, -28) RGC-iN cultures and days 6, 14, and 21 (D6, -14, -21) CTLs. **c** Venn Diagram showing overlapping genes at each time point (normalized count > 10). **d** POU4F1-mNeonGreen/POU4F2-tdTomato dual reporters in RGC-iNs at day 6. **e** Cryosectioned day 45 organoids exhibiting POU4F2-tdTomato + /POU4F1-mNeonGreen+ reporter expression. **f** Heatmap indicating the expression of selected markers for housekeeping (HKG), pluripotency, NAIP2 cassette, photoreceptor (PRs), glial, RGC, inhibitory, excitatory, and pan-neuronal genes. **g** Line graph showing the RGC transcription factors (TFs) NEUROG2, ATOH7, ISL1, POU4F1, and POU4F2 expression at each time point. **h** Line graph for selected neurotrophin receptors and immunostaining of day 6 RGC-iNs with pCREB after 30 min BDNF treatment. **i** Heatmap highlighting ion channel genes expressed at each time point. **j** UMAPs of RGC-iNs colored by time point (top) and Louvain clusters (bottom). **k** UMAPs of RGC-iNs colored by pan-neuronal marker gene expression (top) and RGC-specific marker genes (bottom). **l** Heatmap showing normalized expression of various RGC-subtype marker genes in each cluster of RGC-iNs across day 7, 14, and 21 time points. *P < 0.05, **P < 0.01, ***P < 0.001, ****P < 0.0001, ns *P* > 0.05, *n* = 3 for PSCs, *n* = 4 for all other samples. Error bars **g**, **h** = SEM. Scale **d** = 50 µm, **e** = 200 µm, **e**, inset = 50 µm, **h** = 85 µm.

To evaluate temporal changes in gene expression, we performed bulk RNA-seq for RGC-iNs up to 4 weeks (Fig. 5a) and controls for 3 weeks (Supplementary Table 1). RGC-iNs and controls (*n* = 4 each) showed a diverging pattern of global gene expression that clustered as distinct groups at each time point (Fig. 5b). A Venn diagram highlighted distinct DEGs for each time point (Fig. 5c). To visualize whether *POU4F2* is part of a larger RGC transcriptional network, we generated dual color p2A-mNeonGreen and p2A-tdTomato reporters for the endogenous *POU4F1* and *POU4F2* loci, respectively, and stably integrated TetO-*NAIP2* as previously described. After observing no constitutive reporter expression in PSCs, we began *NAIP2* induction and saw considerable overlap in POU4F1 and -2 expression (Fig. 5d; yellow arrow), similar to the pattern in retinal organoids (Fig. 5e). Upregulation of POU4F1-p2A-mNeonGreen in *NAIP2*-induced cells indicated that POU4F1 and -F2 might be co-regulated and that RGC-iNs were heterogeneous. After dox removal, *NEUROG2* and *ATOH7* were downregulated, while the later RGC markers *ISL1* and *POU4F1* remained constant (Fig. 5f, g). The fact that ISL1 and POU4F2 were expressed was not just a reflection of the dox-induced transgenes. Alignment of the RNA-seq data to the NAIP2 transgene cassette (TetO-NEUROG2-p2A-ATOH7-p2A-ISL1-p2A-POU4F2) or to mRNA untranslated region sequences (UTRs) not part of the transgene, showed that both exogenous and endogenous transcripts were increased (Supplementary Fig. 3g–m). For reasons that are unclear, *POU4F2* was highly expressed at 1 week, gradually decreased at days 14 and 21, then began to rebound by day 28 (Fig. 5g). Other RGC markers (e.g., *POU4F1*, *SNCG*, *NEFL*, and *INA*) were expressed throughout differentiation. RNA-seq alignments between UTRs and temporal RNA-seq samples confirmed that endogenous *ISL1* and *POU4F2* transgenes were expressed even after dox withdrawal (Supplementary Fig. 3k–m). The SoxC subfamily TFs, including *SOX4*, *−11*, and *−12*, which are important for axon guidance and RGC specification, were also abundant^17^(Supplementary Fig. 7). Together, these data indicated that RGC transcriptional networks were regulated by *NAIP2* overexpression.

To rule out the possibility that RGC-iNs included other cell types, we analyzed other cell-type markers by bulk RNA-seq (Fig. 5f). As expected, RGC markers (e.g., *POU4F1*, *POU4F2*, *SNCG, NEFL, INA*, and *ISL1*) and pan-neuronal markers (e.g., *SYN1*, *NCAN*, *NCAM1*, and *RBFOX3* (NeuN)) were well represented between 1 and 4 weeks but glial (e.g., *S100B, PDGFRA, OLIG2, GFAP, RLBP1*), photoreceptor (e.g., *RHO, CRX, ARR3, LHX4*), amacrine (e.g., *GAD1, TFAP2B, TFAP2C*), horizontal (e.g., *LHX1, CALB1, PTF1A*), bipolar cell (e.g., *NETO1, VSX2, OTX2*) and PSC markers (e.g., *NANOG*, *DNMT3B*) were not. Together, this demonstrates that RGC-iNs resemble RGCs and not other retinal cell types.

Neurotrophins, which play a vital role in neuroprotection and neural function^32,33^ also showed dynamic expression. The BDNF receptor *NTRK2* (TrkB), which is important for RGC survival, increased over time while *NTRK1* (TrkA) and *GFRA1* each initially increased then decreased (Fig. 5h). To evaluate functional responses to BDNF, we treated RGC-iNs with BDNF (50 ng/ml) and performed immunocytochemistry for phospho-CREB (Fig. 5h). Compared with untreated controls that showed little to no phosphorylation, a 30-min BDNF treatment showed significant CREB phosphorylation, thus verifying that RGC-iNs were responsive to BDNF. Given the apparent expression of other neurotrophin receptors at earlier stages, it is possible that the addition of their corresponding growth factors (NGF, NT3, CNTF, etc.) could increase survival and alter the proportion of existing cells.

RGCs consolidate visual processing and serve as the main conduit of information from the eye to the brain, and discharge patterns of RGCs, which convey visual information, are determined by a complex repertoire of ion channels. In RGC-iNs we detected voltage-gated sodium (*SCN2A*/Nav1.2a, *SCN3A*/Nav1.3a, *SCN3B*/Nav1.3b, *SCN9A*/Nav1.7) and potassium channels (*KCNA2*/Kv1.2, *KCNA3*/Kv1.3, *KCNA6*/Kv1.6, *KCNC4*/Kv3.4, *KCND1*/Kv4.1, *KCND4*/Kv4.3), which are key modulators of excitability and regulate action potential duration/amplitude (Fig. 5i). We also observed elevated levels of inwardly rectifying potassium channels (*KCNJ3*/Kir3.1, *KCNJ6*/Kir3.2, *KCNJ9*/Kir3.3), which alter RGC firing patterns, and high levels of voltage-gated calcium channels (*CACNA1A*/Cav2.1, *CACNA1B*/Cav2.2, *CACNA1E*/Cav2.3) that regulate synaptic output and plasticity; both are indicative of maturating neurons. The relationship between these channels and electrophysiological responses in the retina has been well documented, however, their expression alone does not confirm that these are RGCs since they are also widely expressed across CNS neurons^34,35^.

Neuron function also requires proteins for packaging, docking/release and reuptake of neurotransmitters. Elevated *SNAP25, SYP, SYT1*, and *VAMP2* expression confirmed that such machinery existed (Supplementary Fig. 8a). While RGCs respond to inhibitory input, their main output is excitatory. We verified the presence of the excitatory glutamate synthetic enzyme glutaminase (GLS), which was detected at high levels (Supplementary Fig. 8b–g). GABA signaling, on the other hand, was unlikely since the expression of the GABA transporters *SLC6A1*, -*A11*, and -*A12*, which are needed for GABA packaging into synaptic vesicles, was very low (Supplementary Fig. 8h–j). Moreover, GABA-transaminase (*ABAT*), which degrades GABA, was highly expressed (Fig. 8Sh–j). Unexpectedly, *GAD1*, a GABA synthetic enzyme, was detected, however, given the low transporter levels and high ABAT levels, accumulation of GABA inside the cell is unlikely. SHMT2 and PHGDH, which regulate glycine synthesis and degradation, respectively, were also expressed at all stages (Supplementary Fig. 8k, l), however, *SHMT2* does not necessarily indicate synaptic activity since glycine is also widely used for protein synthesis. The SLC32A1 (VIAAT/VGAT) transporter, which loads GABA and glycine into synaptic vesicles, is a main vesicular phenotype determinant for glycine and this was not detected. Thus, these cells are not likely to be glycinergic. RGCs also respond to a variety of synaptic inputs including glutamate, GABA, and glycine. AMPA (*GRIA2, -3, -4*), Kainate (*GRIK3*), Metabotropic (*GRM3, -8*), and NMDA (*GRIN1, -2A, -2B, -2C*, and *-3A*) type glutamate receptors (Supplementary Fig. 8b–f) were all detected in addition to the inhibitory ionotropic (e.g., *GABRA3*, *GABRB3*) and metabotropic (e.g., *GABBR2*) GABA receptors (Supplementary Fig. 8h, i) and *GLRA2*/*3* and *GLRB* glycine receptors (Supplementary Fig. 8k). Together these data suggested that RGN-iNs were excitatory in nature and expressed receptors for excitatory and inhibitory synaptic inputs.

### Single-cell analysis reveals subtype diversity

Electrical responses and dendritic arborization are two criteria for classifying RGCs, however, molecular signatures from single-cell RNA-seq (scRNA-seq) have also contributed to our understanding of cell diversity^36,37^. To explore RGC subtypes, we performed scRNA-seq at days 7, 14, and 21 using particle-templated instant partition sequencing (PIP-seq)^38^. For day 7 (*n* = 3) we obtained 3440 cells (27,485 median transcripts/cell), for day 14 (*n* = 2) we obtained 1665 cells (39,208 median transcripts/cell) and for day 21 (*n* = 3) we obtained 2380 cells (31,880 median transcripts/cell). Cells from each time points were clustered using the Louvain algorithm and projected by uniform manifold approximation and projection (UMAP) onto 2D space to visualize cell clusters and marker gene expression. Sustained pan-neuronal (e.g., *TUBB3/MAP2/SYT1*) and RGC-enriched (e.g., *STMN2/GAP43/SNCG*) gene expression was detected (Fig. 5j, k). Cell-type marker analysis excluded other retinal cell types, including photoreceptor, glial, amacrine, bipolar and horizontal cells (Supplementary Fig. 9a). Surprisingly, markers for mature bipolar (*OTX2*) and horizontal (*LHX1*) cells, were detected at day 7 but were downregulated thereafter. Additionally, we compared 2-week-old RGC-iNs to publicly available human scRNA-seq datasets^39–41^, including 45-day-old organoid RGCs, 59-day-old fetal RGCs and 40-day-old iPSC-RGCs and found that RGC-iNs integrated well with each of these datasets (Supplementary Fig. 9b).

Analysis of subtype markers illustrated that RGC-iNs expressed both alpha- (e.g., *SPP1*, *PIGO*, *RPP14*) and directionally sensitive (DS) (e.g., *DCX*, *CDH6*, *JAM2*, *GSN*) RGC markers (Fig. 5l). Some of these (e.g., *JAM2, GSN*) showed an upward trend across time which might suggest an increase in DS-like cells. Since many alpha and DS markers were co-expressed in the same clusters, we explored whether adult human retinas from published scRNA-seq datasets also exhibited mixed expression^42,43^. While cells showed clear separation through Louvain clustering in adult retinas, many alpha and DS markers were again shared across clusters (Supplementary Fig. 10a, a1). Similar results were obtained with day 21 RGC-iNs, which are more mature than earlier time points (Supplementary Fig. 10b, b1) suggesting that the alpha- and DS- RGCs classifications in humans may be difficult to resolve with existing data. To explore whether classifications based on morphology might work better, we extracted the top 2000 highly variable genes (HVGs) at day 21 and used a machine learning-based predictive label transfer model to infer subtypes from a previously annotated human retina scRNA-seq dataset^43^. Compared with the adult human retina that had 90% M-type, 5% P-type, 1% ipRGCs and 4% other RGCs with discrete groupings (Supplementary Fig. 10c), RGC-iNs showed ~40% midget (M-type), 13% parasol (P-type), 7% ipRGC and 40% other RGC subtypes with less discrete groupings (Supplementary Fig. 10c1, c2). Since RGC-iNs did not express *OPN4*, the label transfer approach attempted to label ipRGCs based on more subtle gene expression trends and likely overestimates the number of actual ipRGCs.

A final level of analysis was conducted to explore whether RGCs could be categorized as susceptible or resilient to injury; this classification is based on gene expression profiles in mouse RGCs that survive mechanical injury^44^. RGC-iN clusters showed heterogeneous expression of susceptible and resilient markers at day 21 and consistent expression of survival-promoting neurotrophic factor receptors (*GDFRA1*, *NTRK2*, *NTRK3*, and *CNTFR)* in almost all clusters (Supplementary Fig. 10d). Interestingly, many markers ascribed to susceptible or resilient RGCs were often expressed in the same cell clusters which might suggest that these classifications might not be so binary in developing human RGC-iNs. Given that BDNF and GDNF are added as a supplement to neuronal culture medium their presence could reflect preferential survival of those cell subtypes. Intriguingly, *NTRK1* and *NGFR* were minimally expressed in cells from each day 21 cluster. In summary, while temporal bulk RNA-seq confirmed the developmental trajectory of RGC-iNs, scRNA-seq reaffirmed this trend while providing additional insight into the heterogeneity of RGC-iN subtypes.

### Induced RGC neurons are physiologically active

**Fig. 6:**
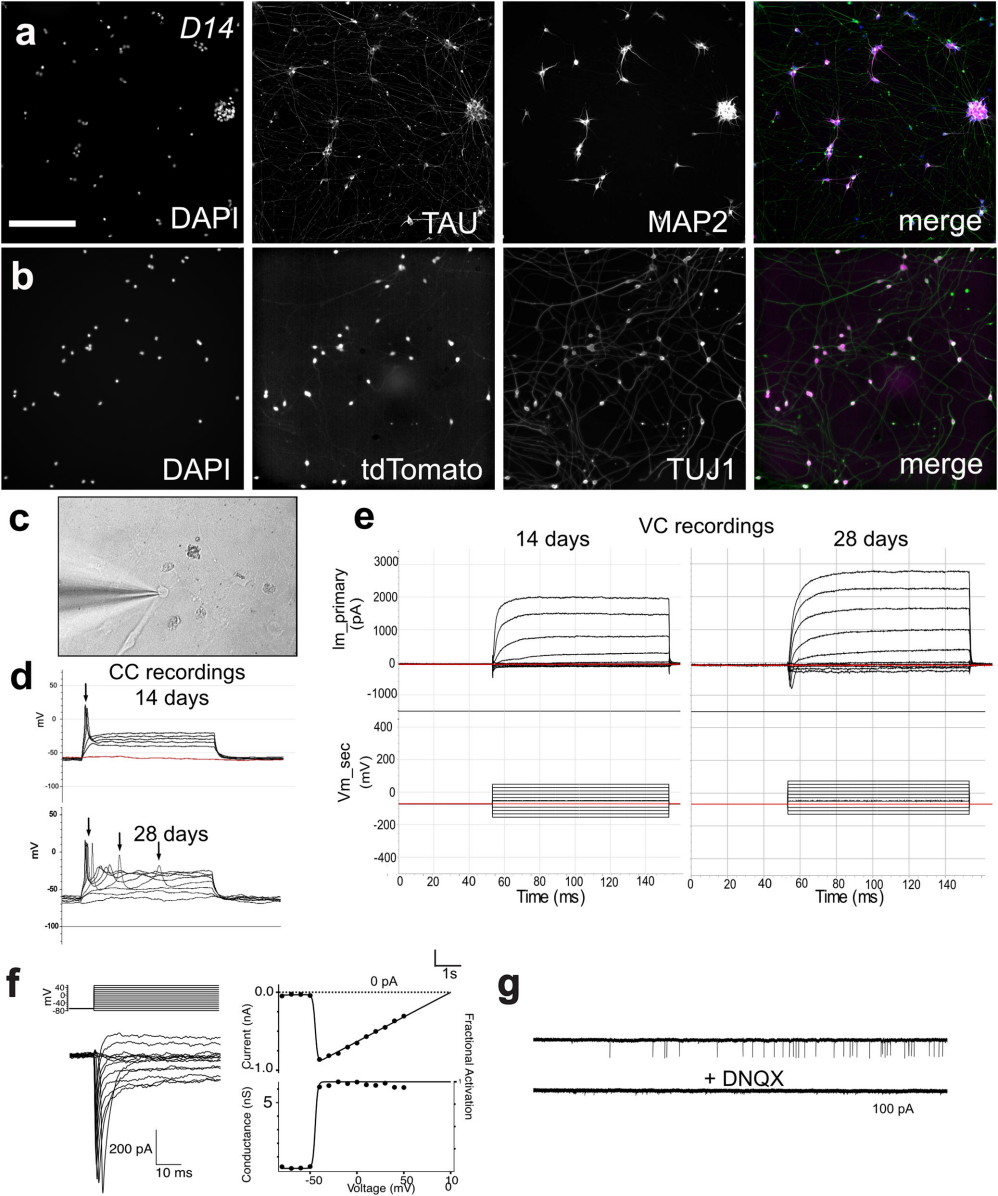
Functional analysis of RGC-iNs. Day 14 (D14) RGC-iNs stained with **a** DAPI, TAU, and MAP2. **b** DAPI and TUJ1 overlayed with tdTomato. **c** Patched D14 RGC-iN. **d** Electrophysiological recordings from RGC-iNs under current clamp showing single action potentials elicited in response to depolarizing current injection as early as 14 days. By day 28, RGC-iNs are shown to exhibit repetitive action potentials. Resting membrane potential = −60 mV. Threshold current = 60 pA. **e** Under voltage clamp, depolarizing voltage steps in RGC-iNs reveal both fast inward Na+ and outward K+ currents, which are increased at day 28 compared to day 14. **f**, left: An example AMPA puff (1 mM) evoked current in a voltage-clamped RGC-iN. **f**, right: I–V plot for AMPA puff evoked currents in RGC-iN (solid symbols), and linear fit (solid line). Traces on the left show time course and double exponential fit to putative sEPSCs. **g** Example of current traces showing spontaneous excitatory post-synaptic currents (sEPSCs) that were blocked by the AMPA/Kainate antagonist DNQX. Scale **a**, **b** = 200 µm.

At two weeks, both TAU and MAP2 showed overlapping expression near the soma of the cell, however, TAU also labeled long projections resembling axons which could indicate that the cells are not yet mature (Fig. 6a, b). We also assessed their electrophysiological function by recording from individual neurons (Fig. 6c). Electrophysiological properties in 2- and 4-week-old RGC-iNs were evaluated using current clamp and whole-cell voltage clamp recordings. Current clamp (Fig. 6d) showed that RGC-iNs were capable of firing action potentials while developing the capacity for repetitive action potential firing over time. Voltage-clamp recordings revealed sodium and potassium currents that were characteristic of RGCs and increased with maturation between 14 and 28 days (Fig. 6e). To examine if RGC-iNs expressed functional glutamate receptors we transiently applied puffs of the agonist AMPA (Fig. 6f) and observed large inward currents with a linear I-V trace reversing around 0 mV in response to AMPA puffs, consistent with the activation of functional GluA2 receptors. Intriguingly, in some RGC-iNs, we observed putative spontaneous excitatory post-synaptic currents (sEPSCs), indicating that the RGC-iNs could form synapses, release neurotransmitters and express functional excitatory neurotransmitter receptors at synaptic contacts. These sEPSCs showed fast kinetics that were blocked by the AMPA/Kainate antagonist DNQX, consistent with AMPA-mediated synaptic events (Fig. 6g).

### Cell death after pharmacological axon injury is prevented by blocking the DLK/LZK and GCK-IV pathways

**Fig. 7:**
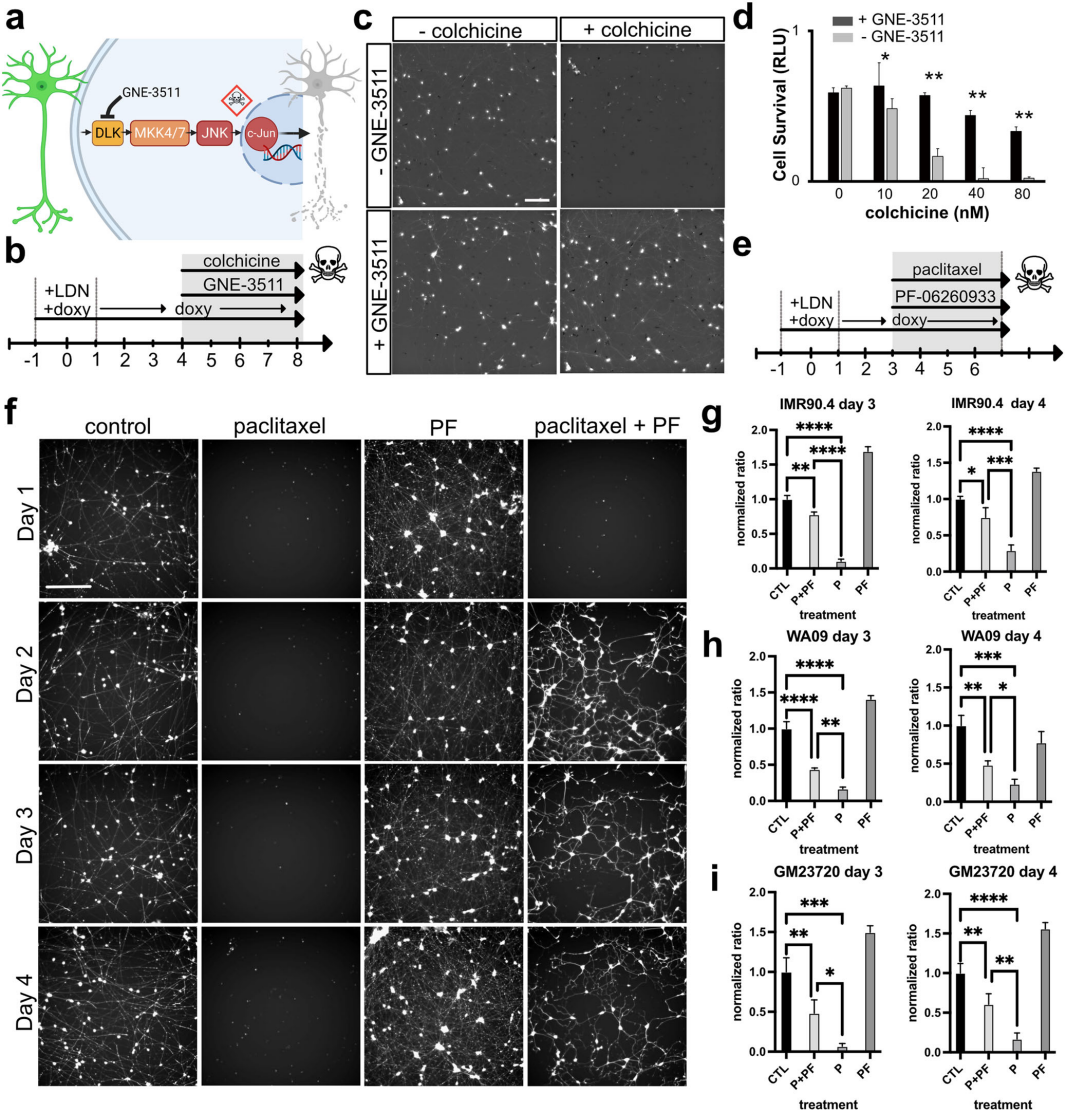
Inhibition of DLK/LZK and GCK-IV pathways protects against neuronal injury. **a** Diagram illustrating a model for blocking DLK-induced cell death. **b** Timeline for RGC-iN differentiation, colchicine-induced injury and neuroprotection with GNE-3511. **c** RGC-iNs treated with or without GNE-3511 (1 μM), 72 h after treatment with or without colchicine (40 nM). **d** Quantification of the CellTiterGlo (CTG) viability assay in the presence or absence of GNE-3511 in combination with increasing doses of colchicine (*n* = 4). **e** Timeline for RGC-iN differentiation, injury from paclitaxel(P), and neuroprotection with PF-06260933(PF). **f** Representative Calcein-AM images after treatment at D1, 2, 3, and 4 with control, paclitaxel, PF-06260933 and paclitaxel + PF-06260933. Quantification of viability using CTG in NAIP2 cells in **g** IMR90.4, **h** WA09, and **i** GM23720 RGC-iNs normalized relative to controls after treatment with P, PF or P + PF (*n* = 3). Scale **c** = 200 µm, **f** = 400 µm. *P < 0.05, **P < 0.01, ***P < 0.001, ****P < 0.0001. Error bars are reported as standard deviation (SD).

A hallmark of neurodegenerative disease, including glaucoma, is axon injury and cell death. To exploit the rapid generation time of RGC-iNs for studies of cell injury and death we leveraged a pharmacological axon injury model involving colchicine, a microtubule destabilizing agent that induces axonal injury and death^45–47^. DLK (MAP3K12) and the related LZK (MAP3K13) signaling pathways are key mediators of RGC injury (Fig. 7a) in addition to mediating responses in other CNS/PNS neurons^48,49^ and their inhibition provides significant protection from RGC death in vitro, induced by colchicine, and in vivo, induced by optic nerve crush^50–52^. To show that RGC-iNs were also susceptible to injury we treated RGC-iNs with colchicine (Fig. 7b). The addition of colchicine led to a dose-dependent increase in cell death, with a complete loss of neurons at 40 nM, measured by the ATP-based CellTiter Glo (CTG) cell survival assay (Fig. 7c, d). Inhibition of DLK/LZK signaling by GNE-3511, a potent DLK/LZK inhibitor, prevented RGC-iN cell loss at all colchicine doses and preserved neuronal morphology as evident by long POU4F2-tdTomato+ neurites.

The GCK-IV kinase family (MAP4K4, MINK, TNIK) also plays an important role in cell death after axonal injury and its inhibition improves survival and neurite outgrowth of mouse and human RGCs in vitro^50^. Furthermore, in the presence of axon injury agents, such as paclitaxel (P), a microtubule stabilizing agent, GCK-IV inhibition effectively prevents axon degeneration in vitro. To extend these findings to human RGC-iNs we injured 3–4-day-old cells with paclitaxel (1 μM) along with PF-06260933 (PF) to inhibit GCK-IV. These were imaged by fluorescence microscopy and quantified by the CTG viability assay after 72 h (Fig. 7e). Massive cell death induced by paclitaxel was observed at days 1, 2, 3, or 4 by microscopy (Fig. 7f). When co-treated with PF-06260933 at the same time points, neurons were significantly protected, except for day 1 which still showed massive cell death. To quantify cell viability, we used the CTG viability assay in three PSC-derived RGC-iNs from three different genetic backgrounds (IMR90.4, WA09, and GM23720). Comparisons between P and P + PF in each background showed that PF was highly neuroprotective (Fig. 7g–i). Furthermore, as previous studies have shown, inhibition of GCK-IV induced robust neurite outgrowth in the absence of injury. This was also observed in RGC-iNs (not shown). Together, these data demonstrated that RGC-iNs are susceptible to multiple forms of axonal injury, engage in established injury signaling pathways and can be protected by blocking those pathways just like actual RGCs, thus establishing this as a rapid and robust model system for studying neurodegenerative signaling.

## Discussion

In the current study, we exploited normal developmental cues that recapitulated retinal development to create highly enriched populations of RGC-like cells. These findings can be summarized as follows: (1) transgene overexpression drove rapid conversion of PSCs into RGC-like neurons, (2) small molecule inhibition of BMP signaling greatly enhanced RGC conversion, (3) RGC-iNs matured in vitro and showed electrical properties resembling those from RGCs and (4) RGC-iNs were susceptible to axonal injury that was effectively blocked by inhibiting the DLK/LZK or GCK-IV kinase signaling pathways.

Control over cell reprogramming with cell-type precision represents an important step in the development of humanized models that facilitate strategies for cell replacement and neuroprotective drug discovery. Reprogramming by TFs, microRNAs^53^ and/or by treatment with small molecules has created several types of neurons, and a recent merging of small molecule dual SMAD inhibition with *NEUROG2* overexpression efficiently produced patterned forebrain neurons^10^. Many TFs involved in RGC development have been identified, however, the minimal number for RGC specification has not. Overexpression of *NEUROG2* and *ATOH7* in differentiating retinal organoids can increase the number of *POU4F2*, *NF145*, and *RBPMS* positive cells and *ATOH7* alone can enhance RGC production in early-stage optic vesicles^54,55^. In mice, Neurog2 mutants have reduced neurogenesis and RGC specification^5^. Conversely, ectopic expression of Isl1 and Pou4f2 during development can promote RGC formation^12^. In addition, several studies have been partially successful in creating RGCs, however, their efficiencies were considerably lower^11,16,56–58^. In one recent study, the combined treatment of *NEUROG2* overexpression and notch inhibition led to RGC-like cells that were used for cell transplantation^59^. Our own approach was quite different in that we overexpressed not one, but 4 TFs necessary for RGCs and combined that with BMP inhibition which enhanced endogenous POU4F2-tdTomato+ signals above that observed for NEUROG2 alone. Based on RNA-seq, significant transcriptional differences existed between NEUROG2 and NAIP2-induced neurons.

The approximate 5 days that it took to generate RGC-iNs was markedly faster than other approaches that generally take upwards of 3–4 weeks^19,60,61^, but as little as 15 days^62^, albeit with much lower efficiency. It is worth noting that our *NAIP2* cassette was designed based on a candidate approach from existing literature, and it is possible that other TF combinations might be as effective for RGC conversion. For instance, in mice Ascl1, Pou4f2, Islet1, and Atoh1 together were recently demonstrated to direct Müller cell conversion into RGCs^1^. For future endogenous repair efforts, other TF combinations may need to be explored to maximize conversion efficiency.

Despite the high efficiency of our RGC-iN generation, not all cells converted into tdTomato+ neurons. While the single CLYBL SHS was tightly controlled as a docking site for gene integration, it may be more susceptible to gene silencing which could contribute to phenotypic heterogeneity between cells. Random integration by piggyBac transposases and lentiviruses could lead to higher levels of TF expression and neural conversion, however positional mutagenesis and epigenetic silencing would need to be addressed. Regardless, methods to prevent gene-silencing, increase protein expression and provide temporal control over individual TFs could be beneficial.

Over 30 RGC subtypes have been identified in mice based on their unique molecular signatures^37,40,63^, however, in humans and other primates there is thought to be less diversity^23,64^. Classifications based on function have led to the recognition of alpha-, DS-, and ip- RGC subtypes, while differences in morphology/projection have led to the identification of midget (M-type), parasol (P-type), and ipRGC subtypes. Classifying RGC-iNs using alpha- and DS- markers was not possible since most cell clusters expressed both marker types. While adult human RGCs could be identified and grouped as M-type, P-type, and ipRGC subtypes, RGC-iNs could be categorized but did not segregate into groups that were as easy to distinguish. One possibility is that RGC-iNs may need additional time or environmental factors to fully mature. To generate specific RGC subtypes, including ipRGCs, it might also be necessary to further modify the composition of the TF transgene cassettes to match those cells more closely. Overall, our results indicate that direct reprogramming is a highly practical approach for generating human RGC-like cells that are well-suited for studies of development, and neuroprotection and may lead to new approaches for cell transplantation.

## Methods

For a detailed list of key resources see Supplementary Table 2.

### Cells

IMR90.4 iPSCs and WA09 ESCs (WiCell) and GM23720 (female) iPSCs (Coriell) were used for the following study. PSCs were used with approval from the UC San Diego Institutional Review Board. Cells were routinely tested for mycoplasma by PCR.

### Single-cell passage and maintenance of PSCs

Stem cells were maintained antibiotic-free on 1% (vol/vol) Matrigel (MG)-GFR™ (#354230; Corning) extracellular matrix (ECM) coated dishes at 37 °C under hypoxic conditions (10% CO_2_/5%O_2_) in mTeSR1 (Stem Cell Technologies) as previously described^65,66^. Cells were passaged every 4–6 days, with Accutase (#A6964; Sigma) for 10–12 min, dissociated into single cells, quenched with mTeSR1 plus 5 μM blebbistatin (B; #B0560; Sigma), pelleted at 80 × *g* for 5 min, resuspended in mTeSR1+B and plated at 2000 cells per single well of a 12-well plate. After 48 h, cells were fed with mTeSR1 alone.

### Reporter cells

*SIX6*-p2A-h2b-eGFP/*POU4F2*-p2A-tdTomato dual reporter cells in the IMR90 iPSC background were previously made^66^. Reporter lines in two additional genetic backgrounds were made by incorporating *POU4F2*-p2A-tdTomato or *POU4F2*-p2A-h2b-mNeonGreen into GM23720 (Coriell) and WA09 (WiCell) lines, respectively. A dual POU4F1-p2A-mNeonGreen/POU4F2-p2A-tdTomato reporter was made in the IMR90.4 background to evaluate the overlap between POU4F1 and POU4F2+ in RGCs.

### Plasmid preparation for transfection

For routine growth of plasmids, we used Stable *E. coli* cells (#C3040I; NEB) grown at 30 °C to reduce unwanted recombination. DNA for transfection was prepared using the endotoxin-free PureLink HiPure Plasmid Midiprep Kit (K210004; Invitrogen) or ZymoPURE II Plasmid Midiprep Kit (D4200; Zymo Research). Purified plasmids were resolved by agarose gel electrophoresis to rule out RNA or genomic DNA contamination. PureLink RNA Mini Kit (#12183020; Thermo Fisher Scientific) was used to purify total RNA to be used for subsequent cDNA synthesis.

EnAsCas12a-HF1-*CLYBL* plasmid for human *CLYBL* gene targeting. To make the pY026-EnCas12a CLYBL-T1 plasmid for targeting the *CLYBL* site we modified the pY026 plasmid (Addgene #84741)^67^ by introducing E174R, N282A, S542R, and K548R high-fidelity mutations into AsCas12a(Cpf1)^68^ using site-directed mutagenesis. Targeting by EnCas12a used a caged tRNA (catRNA) approach made by adding a 24 bp *CLYBL* gene targeting cRNA sequence (ACTTCCTTACTGTTAACTTCCATA) after a U6-tRNA-direct repeat sequence cassette. An additional direct repeat sequence was positioned 3’ to the CLYBL cRNA before the terminator.

### Transgene donor plasmids for expression cassette integration

To drive transgene expression, we built a custom hybrid plasmid (*CLYBL*-TetO) including homology arms flanking the *CLYBL* safe harbor site, a 6X third-generation (3G) Tet promoter and a BGH polyadenylation signal. Adjacent to this, a Cbh-TagBFP2-p2A-zeocin-SV40 polyadenylation signal was inserted within the *CLYBL* homology arms for selection. For transgene expression, *NEUROG2*, *ISL1* and *POU4F2* sequences were PCR amplified from cDNA made from pooled day 18–45 optic vesicles prepared as previously described^65^, while *ATOH7* was amplified from genomic DNA. For cDNA synthesis, total RNA was isolated with a Quick-RNA Miniprep Kit (R1054; Zymo Research) and reverse transcribed using SuperScript IV reverse transcriptase (Thermo Fisher Scientific; #18090050) according to the manufacture’s recommendations. Single-stranded cDNA was PCR amplified with Phusion Flash Polymerase (#F548L; Thermo Fisher Scientific), purified using a DNA Clean & Concentrator-5 column (#D4013; Zymo Research) and cloned into the hybrid donor backbone using Gibson assembly with HiFi DNA Assembly Mastermix (#E2621S; NEB) or homemade Gibson assembly mix. A 2X homemade Gibson stock mix was prepared by mixing 405 µl’s Isothermal Start Mix [1.5 g PEG8000, 3 ml 1 M Tris-HCl pH 8, 150 µl 2 M MgCl_2_], plus 25 µl 1 M DTT, 20 µl 25 mM dNTPs, 50 µl 50 mM NAD+, 1 µl T5 exonuclease, 31.25 µl Phusion High Fidelity DNA polymerase, 250 µl Taq Ligase and 467.75 µl nuclease-free ddH20 (final vol. = 1250 µl’s). To remove unwanted parental plasmid, Gibson products were digested with DpnI enzyme (#R0176L; NEB) in rCutsmart buffer overnight at 37 °C and inactivated at 80 °C for 20 min. Tet-inducible transgene expression was possible by rtTA integrated at the AAVS1 site (Addgene #60843). Individual transgene expression donors are referred to as CLYBL-CBX3-Cbh-zeo-TetO-NEUROG2, -TetO-ATOH7, -TetO-ISL1, and -TetO-POU4F2. For polycistronic expression of different transgenes, including for *NEUROG2*, *ATOH7*, *ISL1*, and *POU4F2* (TetO-*NAIP2*) together, we used multiple porcine teschovirus-1 2A (p2A) peptide sequences differing in nucleotide, but not amino acid sequences (p2A_a, p2A_b, p2A_c), to prevent spurious recombination. This plasmid with all 4 TFs is referred to as CLYBL-CBX3-Cbh-zeo-TetO-NAIP2. Plasmids were transformed into chemically competent Stable *E. coli*, mini-prepped with a ZymoPURE Miniprep kit (#D4210; Zymo Research) and Sanger sequenced (Eurofins Genomics). Plasmids described here are publicly available through Addgene (#202760, 202761, 202762).

### Transient transfection of Tet-inducible overexpression PSCs

In transient transfection, the *NAIP2* cassette enters the cell but is not stably integrated into the genome. Briefly, when the stem cell colonies were at a high density, they were single cell passaged with Accutase. Approximately 100,000 to 200,000 cells were used per transfection and electroporation was carried out in 10 µl of R-buffer containing 3 µg Tet-inducible expression donor vector using a Neon transfection system (MPK5000; Invitrogen) with the following settings (1300 V, 20 ms, 1 pulse). Cells were plated onto 1% Matrigel-coated plates in mTeSR1 with 5 μM blebbistatin and 1 μM of dox (doxycycline; Sigma #D5207) and imaged on a Leica DMi1 inverted microscope for the first week.

### Generation of stably integrated Tet-inducible overexpression PSCs

For stable integration of Tet-inducible overexpression constructs we transfected 3 µg *CLYBL*-TetO donor plasmid, containing different transgene combinations, and 1 µg of the pY026 EnAsCpf1(Cas12a) CLYBL-T1 catRNA plasmid into stem cells that were previously engineered to include endogenous SIX6-p2A-h2b-eGFP and POU4F2-p2A-tdTomato^66^ reporters and constitutive expression of rtTA-M2 integrated at the AAVS1 locus^69^. Transfection was carried out as above, except that dox was not included, and cells were maintained in mTeSR1 with zeocin (200 µg/ml; InvivoGen #ant-zn-1p) for ~2 weeks to obtain cells with stable integration. Integration was verified by Sanger sequencing (Supplementary Fig. 11).

### Clonal selection of induced neuron PSC lines

For isolating clonally derived hPSCs that exhibit robust neural conversion, zeocin-selected hPSCs were single-cell passaged at a density of 5000 cells/well of a 6-well plate. After colonies had formed, they were lifted from the plate using a cell scraper (#89260; VWR) and transferred into a polystyrene 35 mm petri dish. In a sterile laminar flow hood, individual colonies were picked using a P20 micropipette under a brightfield inverted microscope (Leica DMi1) at 4x magnification and added to separate wells of a 48-well plate. PSC colonies were grown until confluency and replica plated to maintain one plate as hPSCs and the other for differentiation. Clones exhibiting the greatest neuronal conversion based on morphology (Supplementary Fig. 2), were expanded for subsequent studies.

### 3D retinal organoid differentiation

To verify that modified cells retained the ability to differentiate into 3D retinal organoids we generated optic vesicles as previously described with minor modifications^66^ for Fig. 1 and^70^ for Fig. 5. Cell culture medium used for differentiation was as follows: BE6.2-NIM (B27 + E6 at 2X concentration) (neural induction medium) consists of DMEM (Invitrogen #11965) supplemented with 1% B27 vitamin A (-) (Invitrogen #12587010) and 2X E6 supplement (38.8 mg/L insulin (#11376497001; Roche), +128 mg/L L-ascorbic acid (Sigma #A8960), 28 μg/L sodium selenite (Sigma #S5261), 21.4 mg/L holo-transferrin (Sigma #T0665) and 38.8 mg/L NaHCO3). LTR (Long-Term Retina) medium was a 3:1 mix of DMEM:F12 (Invitrogen #11965, #11765) supplemented with 1% B27 (Invitrogen #17504044), 10% heat-inactivated qualified-grade FBS (Invitrogen #16140071), 1 mM sodium pyruvate (Invitrogen #11360), 1xNEAA (#11140; Invitrogen), 1xGlutaMAX (#35050061; Invitrogen) and 1 mM taurine (Sigma #T8691). For optic vesicle induction, PSCs maintained in mTeSR1 were used to initiate serum-free forced aggregates. Stem cells were passaged with a longer Accutase incubation for 12 min and 1000 cells in 50 μl of mTeSR1+B were seeded per well into polystyrene 96-well U-bottom plates (Greiner #650180). Aggregates were transitioned to BE6.2 medium by adding 50 μl + 2% MG on day 1 and 1% MG each day thereafter. On days 4–8, a 50% medium exchange (100 μl) was performed daily and every other day thereafter. The medium contained 1% (v/v) MG and 3 μM of IWR-1e (WNT inhibitor; EMD Millipore #681669) from days 1–6. For long-term maintenance, vesicles were treated with 300 nM SAG (Smoothened agonist; EMD Millipore #566660) from days 8–12 to enhance retinal induction and LTR + SAG from days 12–18. In addition, organoids were transferred at day 10 to 15 ml conical tubes, rinsed 3 times in HBSS to remove Matrigel and resuspended in BE6.2 + SAG. Optic vesicles were excised from the larger organoid structures with sharpened tungsten needles between days 10–14 and maintained at a fixed density limited to 40 vesicles per 10 cm dish. To increase survival and differentiation, 500 nM all-trans retinoic acid (ATRA; Sigma #R2625) was added to LTR medium from day 20.

### Direct conversion of patterned induced neuron (iN) and induced neuron-RGCs (RGC-iN)

See protocols.io (dx.doi.org/10.17504/protocols.io.14egn2pqzg5d/v2) for a detailed protocol. Briefly, PSCs maintained in mTeSR1 under 5%O_2_/10%CO_2_ were pretreated overnight with 100 nM LDN-193189 (Sigma #SML0559) and 1 μg/ml doxycycline (Sigma #D5207) to activate Tet-ON TF expression cassettes. Cells were dissociated into a single cell suspension with Accutase for 12 min at 37 °C, quenched with mTeSR1, pelleted at 80 × *g* for 5 min, resuspended in neural induction medium (NIM) with 5 μM blebbistatin and 1–2 μg/ml of dox and passed through a Falcon 35 μm cell strainer (Falcon #352235). NIM consists of DMEM/F12 (Gibco #11330032), N2 supplement (Gibco #17502048), 1x non-essential amino acids (NEAA; Gibco #11140050) and GlutaMAX (Gibco #35050061) all from Life technologies. Cells were plated at ~25,000 cells/well in TC-treated 12-well plates (7000 cells/cm^2^) additionally coated with 0.2 mg/ml poly-L-ornithine (PLO; Sigma #P3655, rinsed 3x in sterile cell culture grade water and coated a final time with 1% GFR Matrigel (Corning #354230). After 2 days, cells were fed by the addition of 1 ml of fresh NIM plus dox and 1x CultureOne supplement (Gibco # A3320201) and every other day thereafter with a one-third medium exchange. For cultures beyond 6 days, cells were transitioned to BrainPhys (StemCell Technologies #05790) plus B27 supplement^71,72^, 50 ng/ml BDNF (Qkine #Qk050), 10 ng/ml GDNF (Qkine #Qk051), and 10 mM nicotinamide (NIC; Sigma; #72340). To reduce unwanted flat cells, we initially treated neurons with bromodeoxyuridine (BrdU), a thymidine analog known to block the cell cycle^73^, but this was toxic at the antimitotic dose of 10 μg/mL. CultureOne^TM^ supplement reduced flat cells and maintained neuronal viability; thus, it was used for subsequent experiments. Interestingly, CultureOne did not prevent glia-like control cells from forming confluent monolayers. PSCs can be grown in normoxia or hypoxia but for practical reasons, normoxia is more common. Oxygen and CO_2_ can also influence retinal cell differentiation^74,75^. For experiments requiring normoxia, we first transitioned PSCs into normoxia growth conditions for several weeks. For induction, cells were treated with 100 nM LDN-193189 /dox or 100 nM LDN-193189/10 μM SB-431542/dox on days −1 and day 0 (Supplementary Fig. 1).

### Click-iT EdU assay

The Click-iT EdU Cell Proliferation Kit for Imaging, Alexa Fluor 647 dye (ThermoFisher; C10340) was used according to the manufacturer’s recommendations. For the day 0 time point (D-1), CTL, NAIP2, and NEUROG2 stem cells were treated with 1 μg/mL dox and 100 nM LDN for 24 h, then treated with 10 µM EdU for 45 min. Cells were then fixed in 4% paraformaldehyde (PFA; Electron Microscopy Sciences #15710) for 30 min at room temperature and then stained with Alexa Fluor 647 azide and the nuclear counterstain DAPI. For the day 2 time point, CTL, NAIP2, and NEUROG2 cells were differentiated according to the RGC-iN direct conversion protocol. On day 2, approximately 72 h after the addition of dox, the same EdU assay was performed.

### Immunocytochemistry

Cells were fixed in 4% paraformaldehyde (PFA) in 0.1 M phosphate (Sorensen’s) buffer and 5% sucrose for 25 min or with 2% PFA for 45 min, rinsed three times in PBS and incubated for 30 min in blocking/permeabilization buffer containing 5% normal horse serum (NHS) and 0.25% Triton X-100 (Sigma Aldrich #T8787) in 1X PBS. Samples were then incubated overnight at 4 °C with 1:50 mouse monoclonal anti-ISL1 (39.4D5) and 1:250 mouse anti-PAX6c; (both from DSHB), 1:1000 chicken polyclonal anti-TAU (PhosphoSolutions; #1998-TAU), 1:1000 chicken polyclonal anti-MAP2 (Phosphosolutions; #1100-MAP2), 1:500 mouse monoclonal anti-MAP2 (BioLegend, #801807), 1:1000 mouse monoclonal anti-TUJ1 (Covance; #MMS-435P), 1:100 mouse monoclonal anti-BRN3A and 1:100 goat polyclonal anti-pan BRN3 (Santa Cruz; #sc-8429, #sc-6026), 1:100 rabbit polyclonal anti-GLAST (Novus; #NB100-1869) and 1:50 rabbit monoclonal anti-VIM (Abcam; #ab92547), 1:250 rabbit monoclonal anti-pCREB (Cell Signaling Tech; #9198) in PBS containing 2% normal horse serum (NHS) and 0.25% Triton X-100. Secondary antibodies were anti-mouse/rabbit/goat IgG’s (H + L) coupled to Alexa Fluor-488 (Invitrogen, 1:1000), or chicken IgY Alexa Fluor Plus 647 (Invitrogen, 1:2000). 10 μg/ml 4’,6-diamidino-2-phenylindole (DAPI; Roche; 10236276001) was used to visualize cell nuclei and sections were processed without primary antibody as controls. Images were acquired with an ImageXpress Micro Confocal High-Content Imaging System, pseudocolored and merged in ImageJ. Adjustments in brightness and contrast were made using ImageJ (NIH) and/or Affinity Designer (Serif Ltd.). Images for phospho-CREB immunostaining (Fig. 5) were acquired with the same exposure times and processed in ImageJ for brightness and contrast with identical settings.

### Microscopy and image analysis

Cells were imaged in Brightfield using a Leica DMi1 inverted microscope to visualize neurite outgrowth. Fluorescent images and corresponding brightfield images were acquired using an ImageXpress Micro Confocal High-Content Imaging System (Molecular Devices) equipped with Cy5, Texas Red, FITC and DAPI filter sets. For the quantification of cell numbers (Figs. 2h, 3m, p, q), cells were fixed in 4% PFA as above, rinsed in PBS and nuclear counterstained with 3 µg/ml Hoechst 33342 (Invitrogen; H1399) or 10 μg/ml DAPI (Roche; 10236276001). A custom image analysis module in the MetaXpress software package (Molecular Devices) was used for counting. First, a low threshold mask from the DAPI/Hoechst channel was created to identify all cells and POU4F2-tdTomato+ cells were then identified from the TexasRed channel. The percent of tdTomato+ cells was calculated by dividing the total tdTomato+ cells over Hoechst/DAPI+ objects, then multiplying by 100. Custom module settings for cell counts were as follows: ‘count tdTomato objects’: min. width 8 μm, max. width 13 μm, intensity above threshold 175; ‘count DAPI nuclei objects’: min. width 10 μm, max. width 13 μm, intensity above threshold 1200. Measurement inputs were set at a standard area value of 10 μm. For cell quantification of Fig. 3p, q a similar workflow was used except that TUJ1 immunostaining was imaged in the Cy5 channel using Alexa Fluor 647 conjugated secondary antibodies and TUJ1+ neurons were calculated with respect to Hoechst/DAPI+ cells (panel p) and tdTomato+ cells were simultaneously counted with TUJ1+ neurons (panel q). Statistical analysis: For Fig. 2h, an unpaired student’s t-test was used to establish statistical significance, whereas in Fig. 3m, p, q, significance was established by an ordinary one-way ANOVA (*P < 0.05, **P < 0.01, ***P < 0.001, ****P < 0.0001, ns *P* > 0.05, *n* = 3). Error bars are reported as standard deviation (SD).

### Electrophysiology

Cultured cells were continuously perfused (4 ml/min) with artificial cerebrospinal fluid (ACSF) or Ames’ medium and equilibrated with 5% CO_2_/95% O_2_. Patch electrodes (3–5 MΩ) were pulled from borosilicate glass capillaries and filled with K^+^-gluconate solution having a composition (in mM) of: potassium gluconate 147, KCl 1, CaCl_2_ 2, HEPES 10, EGTA 10, Glucose 10, MgATP 2 and GTP 0.3 (300 mOsm, pH 7.3–7.4) for current clamp and potassium current recordings, as described by ref. ^76^, or Cs-Methansulfonate based internal solution containing (in mM) 90 CsCH3SO3, 20 TEA-Cl, 10 HEPES, 10 EGTA, 10 phosphocreatine disodium salt hydrate, 4 Mg-ATP and 0.4 Na-GTP for sodium and AMPA receptor current recordings. Whole-cell patch-clamp recordings were obtained using a Multiclamp700A (Molecular Devices) and digitized at 20 kHz with either a Digidata 1440 A and pClamp software (Molecular Devices), or an Instrutech ITC-18 (HEKA Elektronik) and custom acquisition software in IgorPro (Wavemetrics). Current clamp and potassium current measurements were performed at room temperature. Sodium current recordings, AMPA current recordings and sEPSC kinetic experiments were performed at 35 °C. For puff experiments, a 50 ms pressure pulse was applied to a standard patch pipette filled with 1 mM AMPA in Ames medium. Pressure pulses were controlled by a custom-built pico-spritzer, controlled by the acquisition software.

### Neuroprotection assays

RGC-iNs were grown on PLO/Matrigel-coated TC plates as described above. For the GNE-3511 survival assay, cells were pretreated with 1 μM GNE-3511 (Genentech) for one day on day 4, subjected to increasing doses of colchicine (0–80 nM) for 72 h and analyzed for viability. For the PF-06260933 survival assay: 2 µM PF-06260933 (Pfizer) with or without 1 μM paclitaxel was added at days 1–4 and analyzed on day 7. For both assays, RGC-iN viability was measured using the CellTiter-Glo (CTG; Promega #G7570) luminescent ATP-based reagent. CTG reagent was added to cells at a 1:2 ratio with existing media, incubated at 37 °C for 20 min and measured using a luminescent plate reader (Tecan Infinite M Plex). Imaging of viable cells was performed with 0.2 µM Calcein-AM (Invitrogen; C3100MP) and 3 µg/ml Hoechst 33342 prior to the CTG assay. For Fig. 7d, significance was established by the student’s t-test (*P < 0.05, **P < 0.01, *n* = 4). For Fig. 7g, h, i, an ordinary one-way ANOVA was used for statistical comparisons with *P < 0.05, **P < 0.01, ***P < 0.001, ****P < 0.0001, *n* = 3. Error bars are reported as standard deviation (SD).

### Bulk RNA sequencing

RNA from undifferentiated PSCs (*n* = 3), untreated controls (CTL) at days 6 (*n* = 5), −14 (*n* = 4), and −21 (*n* = 4), NEUROG2/NA iNs at day 6 (*n* = 4), non-clonally selected NAIP2-nc RGC-iNs at day 6 (*n* = 4), and clonally selected NAIP2 RGC-iNs at days 6 (*n* = 4), −14 (*n* = 4), −21 (*n* = 4), and day 28 (*n* = 4) were extracted using the Quick RNA Microprep Kit (Zymo Research; #R1050) per manufacturer’s instructions. RNA quantity and quality were analyzed using a NanoDrop 2000 (Thermo Scientific) and 4200 TapeStation (Agilent Technologies), respectively. RNA-seq libraries were generated using rRNA-depleted (Illumina RiboZero Plus) samples and the TruSeq RNA Library Prep Kit v2 (Illumina). Sequencing was performed on a NovaSeq 6000 (100 bp paired-end reads, Illumina) at the UCSD Institute for Genomic Medicine (IGM) Core to obtain 25 million reads per sample.

### Bulk RNA-seq analysis

Adapters were trimmed from FASTQ files using TrimGalore! (Galaxy Version 0.6.3) and reads were mapped to the human reference genome GRCh38.p13 using HISAT2 (Galaxy Version 2.2.1). Gene expression levels were quantified using FeatureCounts (Galaxy Version 2.0.1+) to determine counts for each gene and differential expression analysis was carried out between treatment groups (CTL, NEUROG2, NA, NAIP2-nc, NAIP2, and PSC) using DESeq2 (Galaxy Version 2.11.40.6) which determines counts and differential expression via the median of ratios method for normalization (Supplementary Table 1). Principal component analysis (PCA) was performed using DESeq2 (Galaxy Version 2.11.40.6) while hierarchical cluster map analyses were performed using Seaborn (version 0.12.0) and Matplotlib (version 3.6.0) in Python (version 3.8.9). Volcano plots were generated using ggplot2 (Version 3.3.5) with RStudio (running R version 4.1.2). Venn diagrams were generated using VennPainter (Version 1.2.0). Scatter plots depicting DESeq2 normalized count values of genes in each treatment group across replicates were made in GraphPad Prism 9 (Version 9.1.1). Differential expression data generated by DESeq2 was then used to identify significantly differentially expressed genes (DEGs) with normalized counts (median of ratios) >10, a Log2 (FoldChange) > 1 and a false discovery rate (FDR) < 0.05. DEGs were then input into DAVID (Version 6.8) and GSEApy (version 1.0.4) to identify pathways as defined by Uniprot, Gene Ontology, Kyoto Encyclopedia of Genes and Genomes (KEGG, Version 99.1), Interpro (Version 86.0) and Simple Modular Architecture Research Tool (Version 9.0) that are significantly differentially expressed. Differentially expressed pathways that were considered statistically significant based upon an FDR < 0.05 were then used to generate a bar chart via Seaborn and Matplotlib which depicts each pathway and its corresponding FDR. Heatmaps of individual pathways identified by DAVID which are known to be involved in retinal development were then used to generate heatmaps via Seaborn/Matplotlib comparing gene expression with each individual replicate of each treatment group based upon the counts value determined by DESeq2. To distinguish between transgene and endogenous expression of *NEUROG2*, *ISL1*, *ATOH7*, and *POU4F2*, we used the built-in short read aligner in Geneious Prime 2022 (Biomatters) to map 1-, 2-, and 3-week-old NAIP2 neuron FASTQ reads to a reference sequence for NEUROG2-p2A-ATOH7-p2A-ISL1-p2A-POU4F2 as well as the 5’ and 3’ up/downstream UTRs for each of the four endogenous TFs. To quantify the number of integrated transgene transcripts, we counted the number of mean aligned reads across all p2A sequences, while for endogenous expression we counted the mean number of reads aligned to the 3’ UTRs since this was not part of the transgene package. Mean aligned reads <5 were assumed to be negligibly expressed. The number of endogenous and transgene aligned reads were plotted as a pie chart in GraphPad Prism.

### Single-cell RNA sequencing (scRNA-seq)

For scRNA-seq experiments, 5000 cells/replicate (*n* = 3 for day 7 and 21, *n* = 2 for day 14) at each time point (day 7, 14, and 21) were concentrated into 5 µL of cell resuspension buffer for PIP-seq (particle-templated instant partition sequencing; Fluent Biosciences), cells were captured, lysed and processed for library preparation as per the manufacturer’s instructions (PIPseq™ T2 3’ Single Cell RNA Kit v4.0). Briefly, mRNA was captured by breaking the stable emulsion via the addition of breaking buffer and cDNA was amplified and isolated from the PIPs by SPRI purification. Quality assessment and quantification of cDNA was achieved using a 4150 TapeStation (Agilent Technologies). cDNAs with values greater than 10 ng and peaks between the acceptable range for the high-sensitivity D5000 ScreenTape (100–5000 bp) were used for library preparation (fragmentation, end repair, A-tailing and adapter ligation) and sample index PCR was performed with unique dual index combinations. The final sequence-ready libraries were assayed for quality and quantity (size analysis of 35–1000 bp fragments via high-sensitivity D1000 ScreenTape) by Tape Station. Sequencing was performed on a NovaSeq 6000 (100-bp paired-end reads, Illumina) at the UCSD Institute for Genomic Medicine (IGM) Core to obtain >200 million reads per sample.

### scRNA-seq analysis

FASTQ reads were processed to obtain the single-cell feature barcode-count matrices using PIPseeker version 2.1 by Fluent Biosciences. Briefly, reads were error corrected using a barcode whitelist and aligned to the reference human genome GRCh38.p13 (GENCODE v40 2022.04, Ensembl 106) using STARsolo (STAR version 2.7.10a)^77^. Aligned/sorted BAM files were used for cell calling using the transcript-count thresholding approach built into PIPseeker. The feature-cell barcode matrix was analyzed using Scanpy (version 1.9.1)^78^. General preprocessing of the count matrices was conducted by filtering out cells expressing less than 200 genes, genes expressed in less than 3 cells, or cells with greater than 5 percent mitochondrial genes. The matrix from each time point was further filtered to eliminate outlier cells with greater than 200,000 transcripts. Post-filtering, there were 3440 cells for day 7 (*n* = 3), 1665 cells for day 14 (*n* = 2), and 2380 cells for day 21 RGC-iNs (*n* = 3). Each cell was normalized to a total transcript sum of 10,000 genes and the counts were log normalized. Principal Component Analysis (PCA) was used for dimensionality reduction, cells were separated into clusters using the Louvain algorithm followed by UMAP generation^79^. These clusters were assessed for marker genes using Scanpy visualization to obtain gene marker dot plots and heatmaps.

To compare the transcriptional profiles of RGC-iNs with published scRNA-seq datasets, we used Scanorama (version 1.7.3)^80^ for panoramic stitching of RGC-iN and published matrices and ComBat^81^ for batch correction. A similar workflow was adopted as above for clustering and visualizing the integrated datasets. We used scvi-tools (version 0.20.3) to utilize predictive label transfer using scANVI, a semi-supervised deep generative model for single-cell data^82,83^. The semi-supervised model was trained on published subtype-labeled data^43^ and then used to predict subtypes in day 21 RGC-iNs.

### Statistical analysis

Results were quantified using Prism 9 (v9.1.1, GraphPad software). Unless otherwise stated, data are presented as mean ± standard deviation (SD) and the following symbols are used to represent *P* values, *P < 0.05, **P < 0.01, ***P < 0.001, and ****P < 0.0001 and non-significant (ns) *P* > 0.05. N represents the number of independent experiments.

### Genotyping of transgene insert

Modified cells were genotyped by PCR to validate transgene insertion into the *CLYBL* locus. Initially, we tested for homo- or heterozygosity by using primers flanking the insert site (CLYBL_GT5_F/CLYBL_GT1_R). An upper band (that varied by insert size) indicated insertion while a lower band of 396 base pairs indicated no insertion (Supplementary Fig. 11). Due to difficulties in amplifying the longer sequences from genomic DNA, the larger band was often missing even though cells were successfully selected by zeocin treatment. In those instances, transgene insertion was validated by PCR with one primer within the transgene cassette and the other flanking the left or right homology arms (left arm: CBX3_GT2_R/CLYBL_HET_4277_F; right arm: H11_Cbh_RuBGH782GT_F/ CLYBL_ GEN_1261_R; Supplementary Table 2). PCR products were Sanger sequenced to confirm the integrity of integrated transgenes.

### Reporting summary

Further information on research design is available in the Nature Research Reporting Summary linked to this article.

### Supplementary information

Supplemetal material

reporting summary

## Data Availability

RNA sequencing datasets are available as raw FASTQ files accessible at the Sequence Read Archive (SRA#; PRJNA885885, PRJNA973095). Processed single-cell feature barcode count matrices are available on GitHub (https://github.com/WahlinLab/Human_RGC-iN). Details about sample accession numbers, names, age, cell line, replicate and cell types can be found in Supplementary Table 3.

## Code availability

For reproducibility, detailed Jupyter notebook files containing all Python code and R scripts are available on GitHub. A list of all the publicly available tools and packages required for analysis is also provided in the repository.
