# Supplementary material for: Human retinal ganglion cell neurons generated by synchronous BMP inhibition and transcription factor mediated reprogramming
Source: NPJ Regen Med. 2023 Sep 29;8:55. doi: 10.1038/s41536-023-00327-x (PMC10541876; doi:10.1038/s41536-023-00327-x)
Supplement: Supplementary file 1 — Supplemetal material [file 41536_2023_327_MOESM1_ESM.pdf]

Human Retinal Ganglion Cell Neurons Generated by Synchronous BMP inhibition and Transcription Factor Mediated Reprogramming.

Devansh Agarwal[1,2‡], Nicholas Dash[2‡], Kevin W. Mazo[2], Manan Chopra[2], Maria P. Avila[2], Amit Patel[2], Ryan M. Wong[2], Cairang Jia[2], Hope Do[2], Jie Cheng[3], Colette Chiang[2], Shawna L. Jurlina[2], Mona Roshan[2], Michael W. Perry[4], Jong M. Rho[5], Risa Broyer[2], Cassidy D. Lee[2], Robert N. Weinreb[2], Cezar Gavrilovici[5], Nicholas W. Oesch[2,6], Derek S. Welsbie[2], Karl J. Wahlin[2*]

Supplementary Materials:

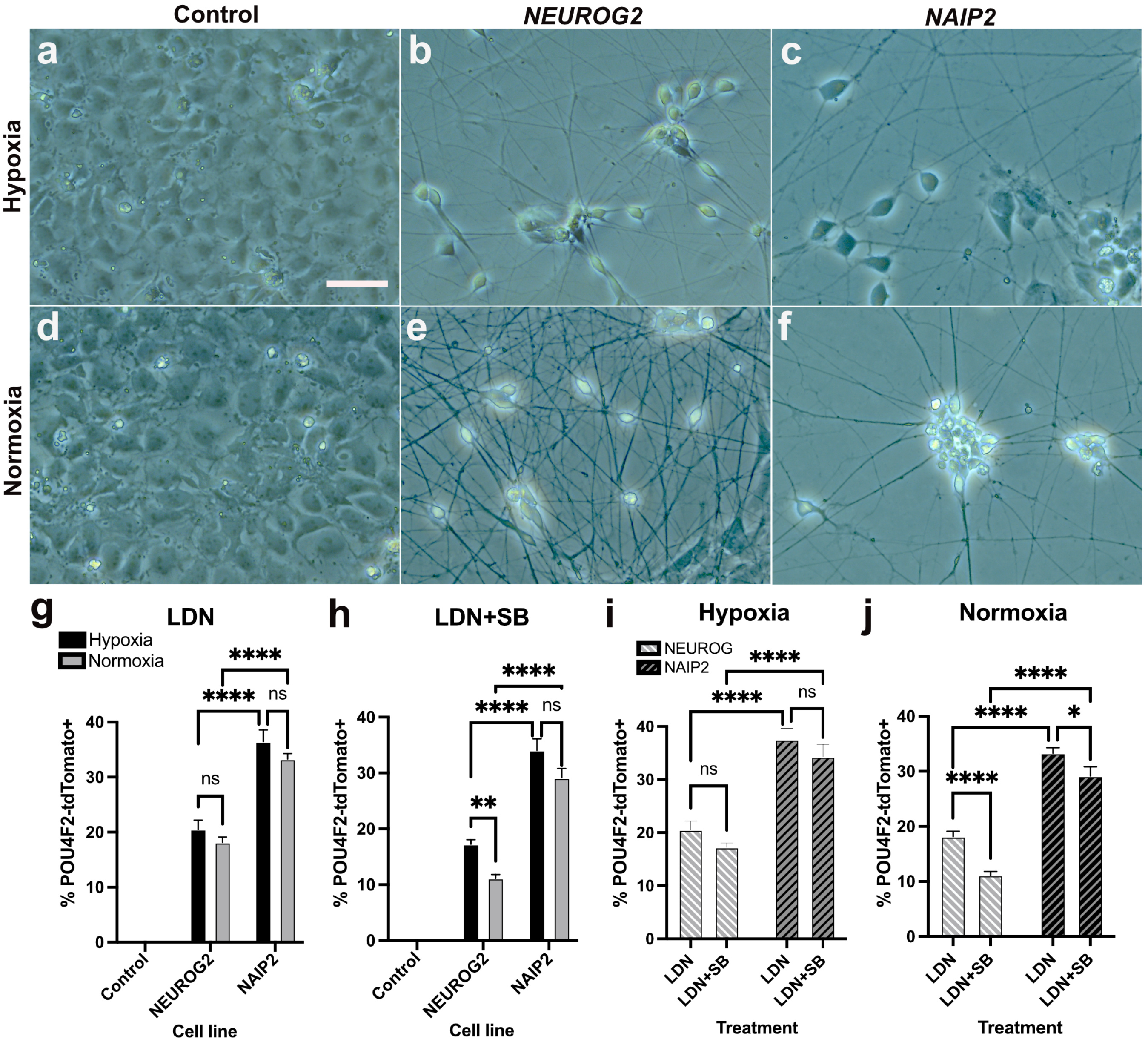

Supplementary Figure 1. ***The role of hypoxia and dual SMAD inhibition on RGC-iNs formation*.** (**a**) Bright-field imaging of D6 normoxia (**a-c**) and hypoxia (**d-f**) conditioned control, NEUROG2, or NAIP2-nc (prior to colony selection) cells after treatment with LDN/dox. (**g**) Evaluation of POU4F2-tdTomato+ cells in control, NEUROG2, NAIP2-nc samples treated with (**g**) LDN or (**h**) LDN + SB431542 in hypoxia and normoxia. (**i**) Comparison between POU4F2-tdTomato+ cells after LDN or LDN193189 plus SB431542 under (**i**) hypoxia or (**j**) normoxia. Statistical significance was determined by ordinary two-way ANOVA with Tukey's multiple comparison and FDR <0.05. (n=3

biological replicates; P values: <0.05*, <0.01**, <0.0001***, <0.00001****; error bars: SEM). Scale=100μm. NAIP2-nc indicates non-clonally selected NAIP2 PSCs.

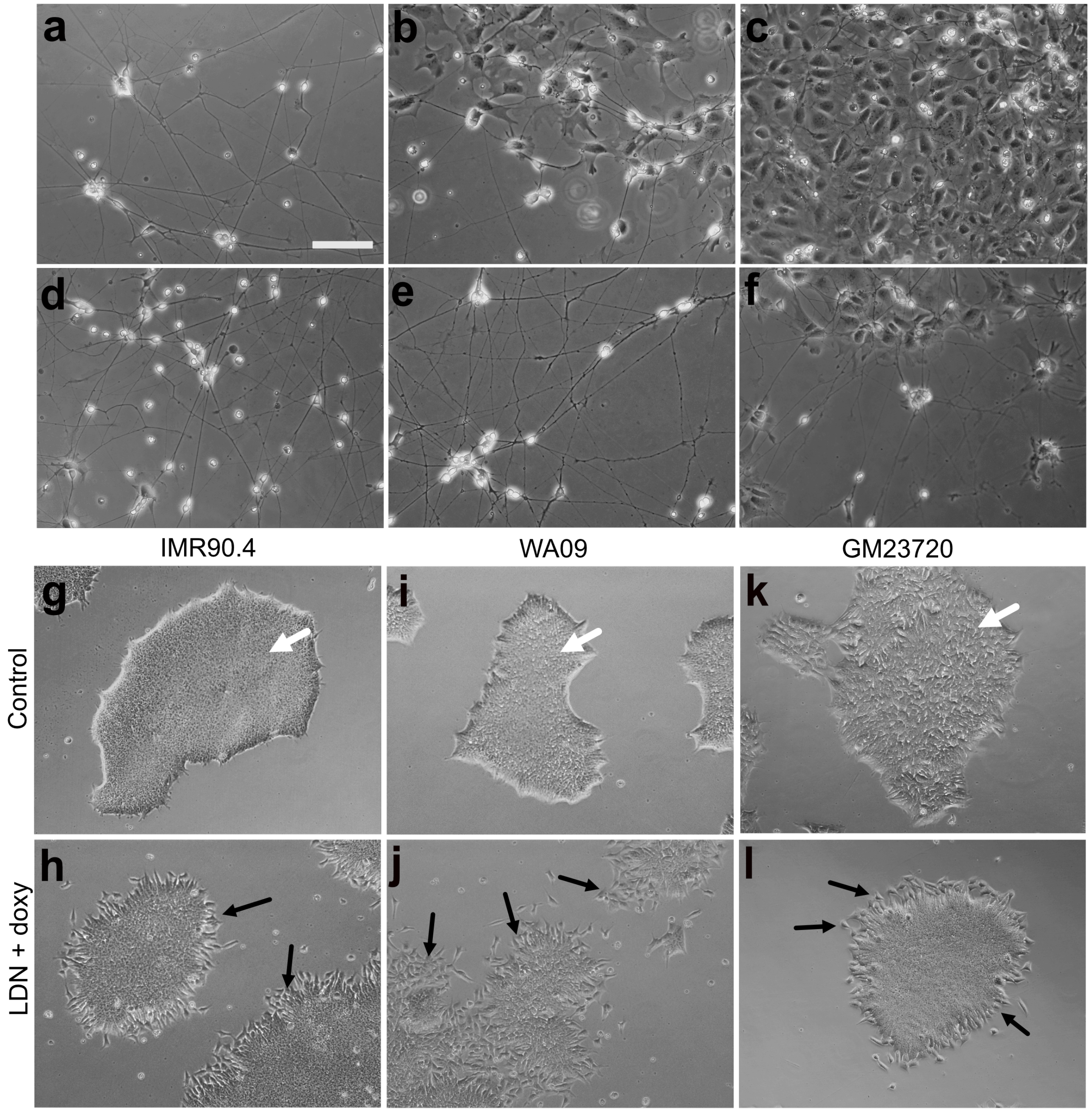

Supplementary Figure 2. ***Clonal enrichment of neuronal producing clones following zeocin selection and manual isolation and confirmation of neuronal induction by LDN/dox.*** (**a-f**) Individual NAIP2 clones in differentiated (NAIP2c1-6) PSCs following zeocin selection and manual clonal selection. Scale=200µm. Morphology of NAIP2 cells in three genetic backgrounds (IMR90.4, WA09, GM23720) in untreated controls (**g,i,k**) and after 24 hours of LDN/dox treatment (**h,j,l**).

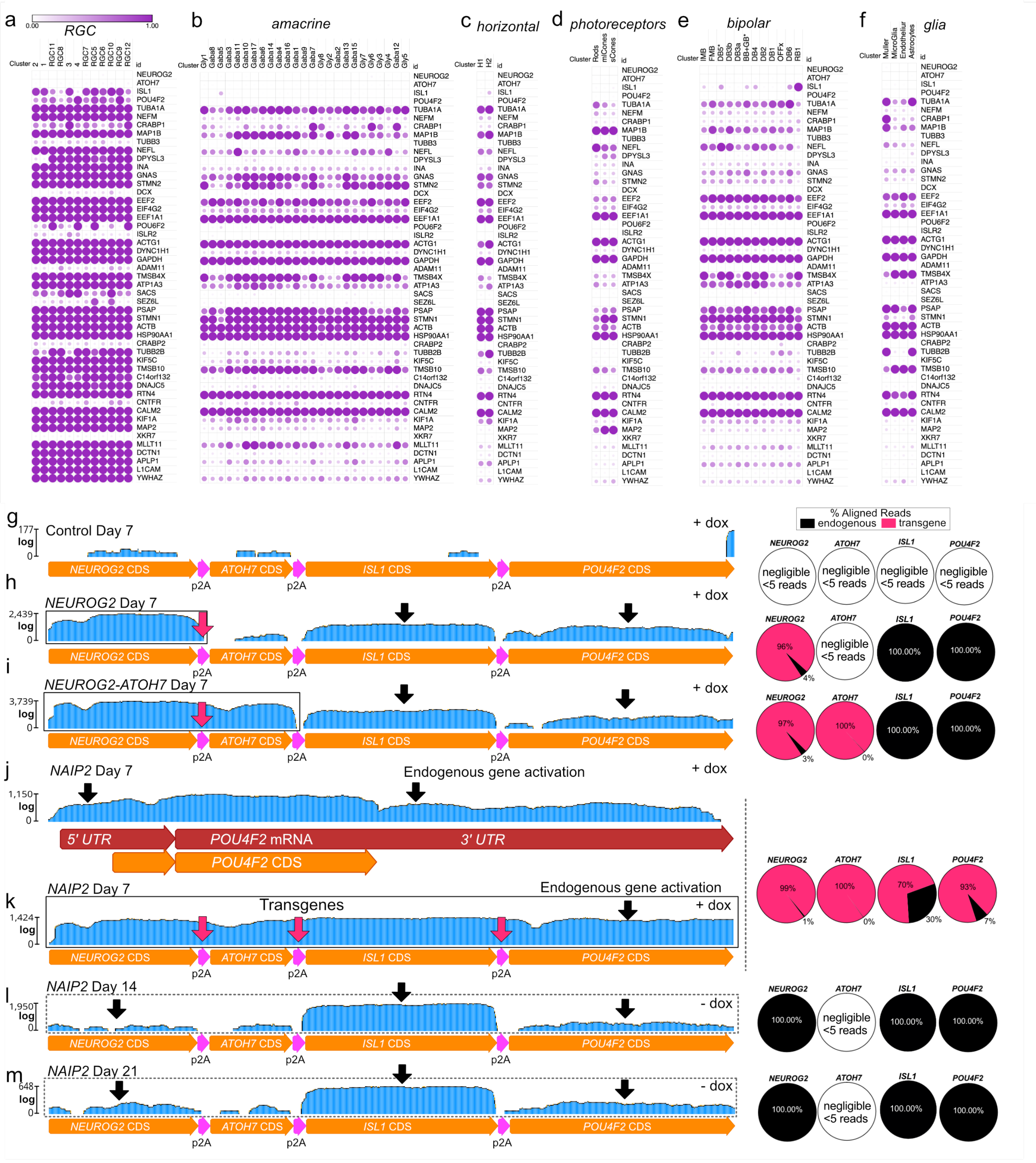

Supplementary Figure 3. ***Endogenous and transgene expression and correlation of RGC-iN bulk RNA-seq to human foveal and peripheral retina RGC cell clusters.*** Correlation of the top 50 RGC-iN expressed genes from bulk RNA-seq on day 7 relative to RGC cell clusters (**a**) identified by scRNA-seq of the human fovea and peripheral retina (https://singlecell.broadinstitute.org/; Yan et al., 2020). Amacrine (**b**), horizontal (**c**), photoreceptor (**d**), bipolar (**e**) and glial (**f**) cell clusters were also presented to demonstrate contrast to RGCs. RNA-seq coverage maps (left) and pie charts (right) illustrating % endogenous versus transgene aligned reads identified using the Geneious RNA-seq aligner with one-week old (**g**) control, (**h**) NEUROG2, (**i**) NEUROG2-ATOH7 cells aligned to the *NAIP2* gene

cassette or (**j**) NAIP2 expressed transcripts aligned to the endogenous *POU4F2* gene. (**k-m**) NAIP2 transcripts from RGC-iNs after 1, -2 and -3 weeks. Black arrows (**j**) indicate endogenous gene expression mapped to the 5 prime or 3 prime untranslated regions, while pink arrows (**h,i,k**) indicate p2A sequences that map to the NAIP2 transgene cassette.

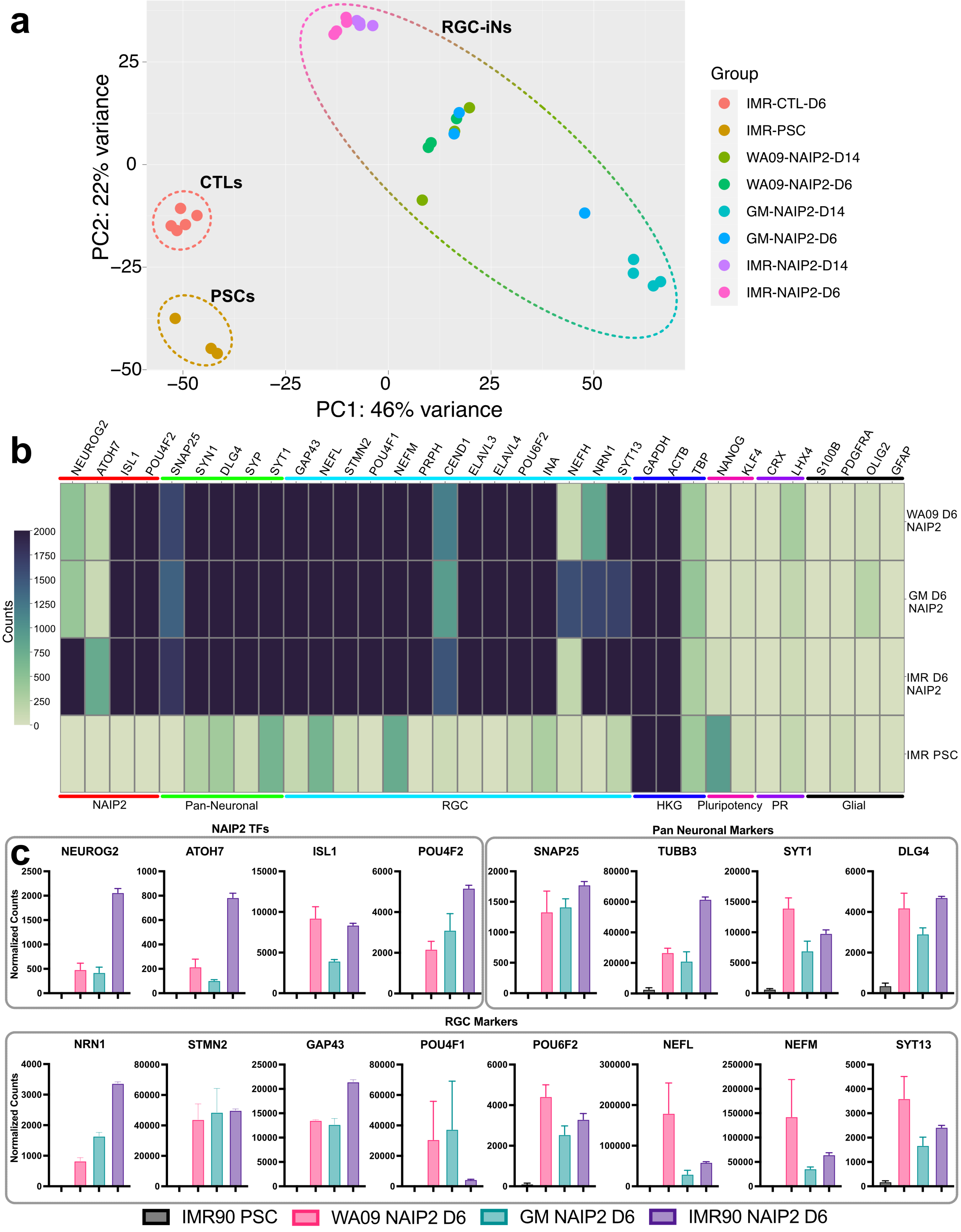
a
RGC-iNs
CTLs
PSCs
PC1: 46% variance
PC2: 22% variance
Group
IMR-CTL-D6
IMR-PSC
WA09-NAIP2-D14
WA09-NAIP2-D6
GM-NAIP2-D14
GM-NAIP2-D6
IMR-NAIP2-D14
IMR-NAIP2-D6
b
NEUROG2 ATOH7 ISL1 POU4F2 SNAP25 SYN1 DLG4 SYP SYT1 GAP43 NEFL STMN2 POU4F1 NEFM PRPH CEND1 ELAVL3 ELAVL4 POU6F2 INA NEFH NRN1 SYT13 GAPDH ACTB TBP NANOG KLF4 CRX LHX4 S100B PDGFRA OLIG2 GFAP
Counts
WA09 D6 NAIP2
GM D6 NAIP2
IMR D6 NAIP2
IMR PSC
NAIP2
Pan-Neuronal
RGC
HKG
Pluripotency
PR
Glial
c
NAIP2 TFs
NEUROG2
ATOH7
ISL1
POU4F2
Pan Neuronal Markers
SNAP25
TUBB3
SYT1
DLG4
RGC Markers
NRN1
STMN2
GAP43
POU4F1
POU6F2
NEFL
NEFM
SYT13
Normalized Counts
IMR90 PSC
WA09 NAIP2 D6
GM NAIP2 D6
IMR90 NAIP2 D6

Supplementary Figure 4. ***Comparison of gene expression profiles among RGC-iNs derived from PSC lines across various genetic backgrounds.*** (**a**) Principal Component Analysis (PCA) plot of PSCs, day 6/14 IMR90-CTL, IMR90-NAIP2, GM-NAIP2, WA09-NAIP2 RGC-iNs. (**b**) Heatmap indicating the expression of selected markers for housekeeping (HKGs), pluripotency, NAIP2 cassette, photoreceptor (PRs), glial, RGC, and pan-neuronal genes. (**c**) Bar graphs showcasing the expression of NAIP2 transcription factors (TFs), pan-neuronal marker and RGC marker genes in IMR90-PSCs, WA09-NAIP2-D6, GM-NAIP2-D6 and IMR90-NAIP2-D6 RGC-iNs (n=3 for IMR90-PSCs/WA09-NAIP2-D6/WA09-NAIP2-D14/GM-NAIP2-D6, n=4 for GM-NAIP2-D14/IMR90-NAIP2-D6/IMR90-NAIP2-D14, n=5 for CTL, error bars=SEM).

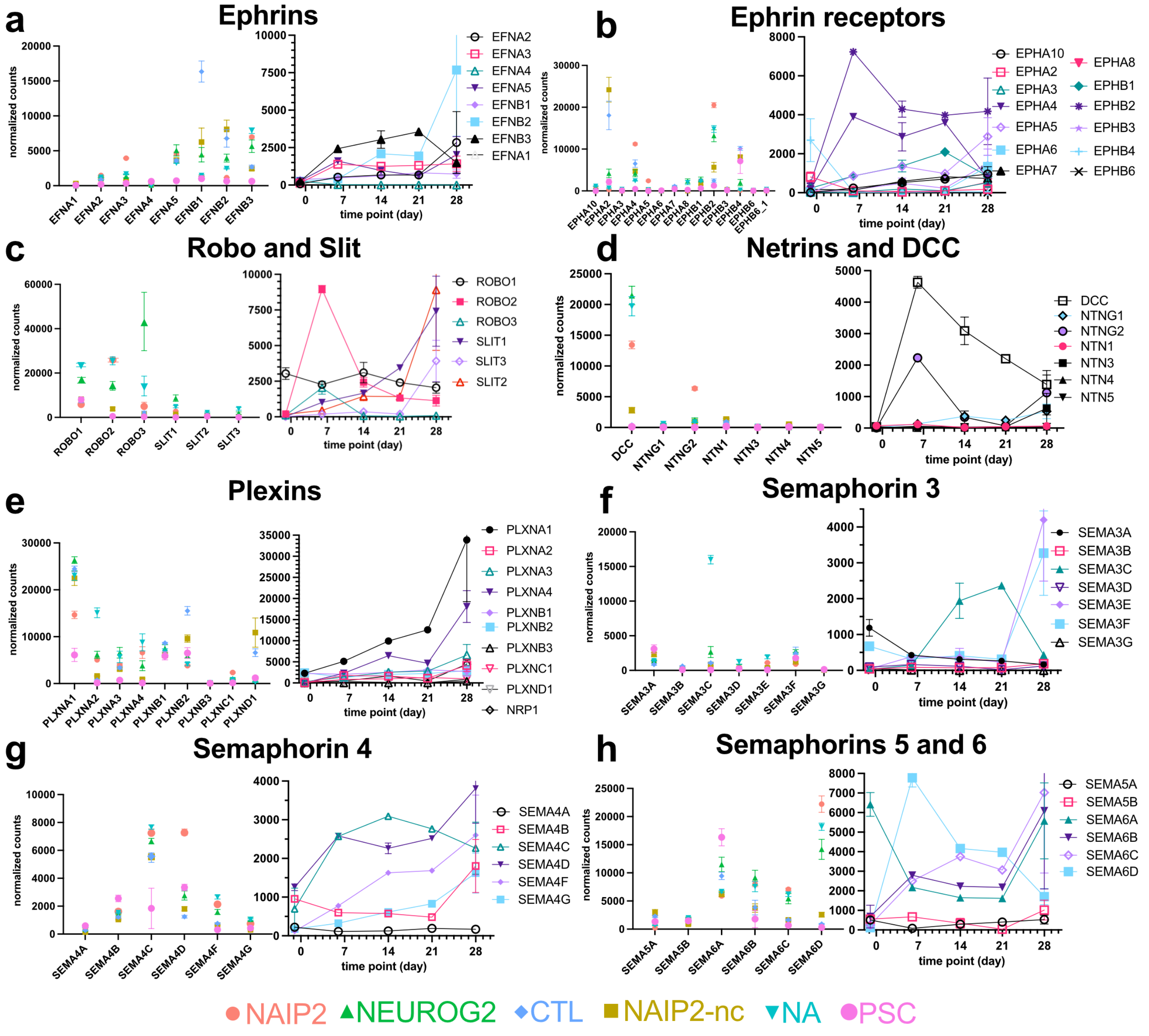

Supplementary Figure 5. ***Axon guidance cue expression in RGC-iNs over time.*** The first plot in each panel (**a-h**) represents bulk RNA-seq normalized counts compared between IMR90.4 NAIP2, NAIP2-nc, NEUROG2, NA, CTL samples differentiated for one week or undifferentiated PSCs. NAIP2-nc indicates non-clonally selected NAIP2 PSCs (n=3 for PSCs, n=4 for NAIP2/NEUROG2/NA, n=5 for NAIP2-nc /CTL, error bars=SEM). The second plot in each

panel (**a-h**) represents bulk RNA-seq normalized counts compared between IMR90.4 NAIP2 iPSCs at 0, 1, 2, 3 and 4 weeks (n=3 for PSCs, n=4 for all other treatment groups, error bars=SEM).

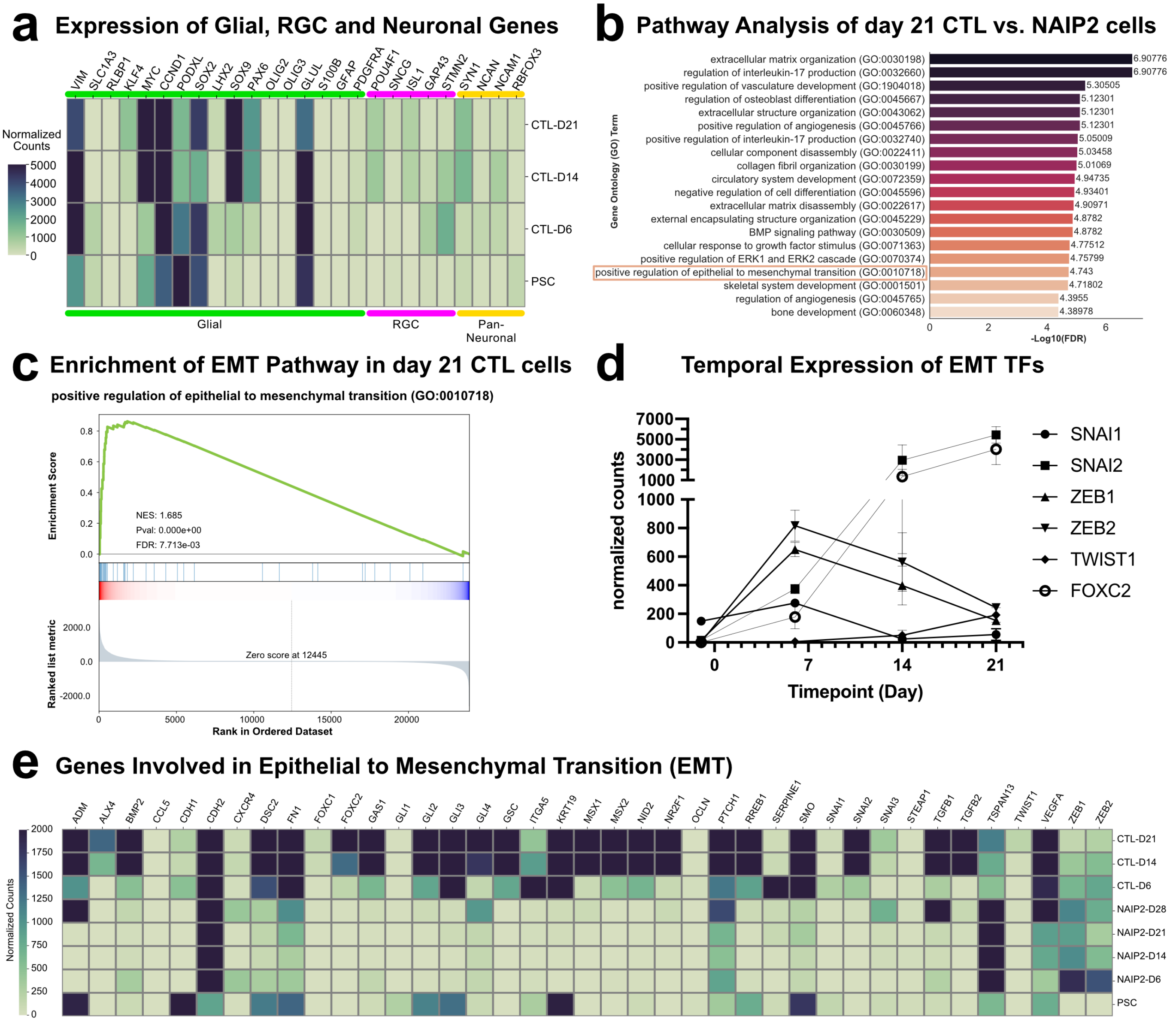

Supplementary Figure 6. ***Gene expression in CTL cells over time.*** (**a**) Heatmap showing expression of glial, RGC and pan-neuronal genes in PSCs, CTL -D6, -D14 and -D21 samples. Gene set enrichment analysis of day 21 CTL versus NAIP2 cells indicating (**b**) bar plot of the top 20 upregulated pathways ranked by –log10(FDR) and (**c**) rank/enrichment of the epithelial to mesenchymal transition (EMT) pathway. (**d**) Temporal expression of EMT transcription factors in PSCs, CTL-D6, -D14 and -D21 samples (n=3 for PSCs, n=4 for CTL-D14/-D21, n=5 for CTL-D6, error bars=SEM). (**e**) Heatmap showing expression of genes involved in EMT in PSCs, NAIP2-D6, -D14, -D21, -D28, and CTL-D6, -D14 and -D21 samples.

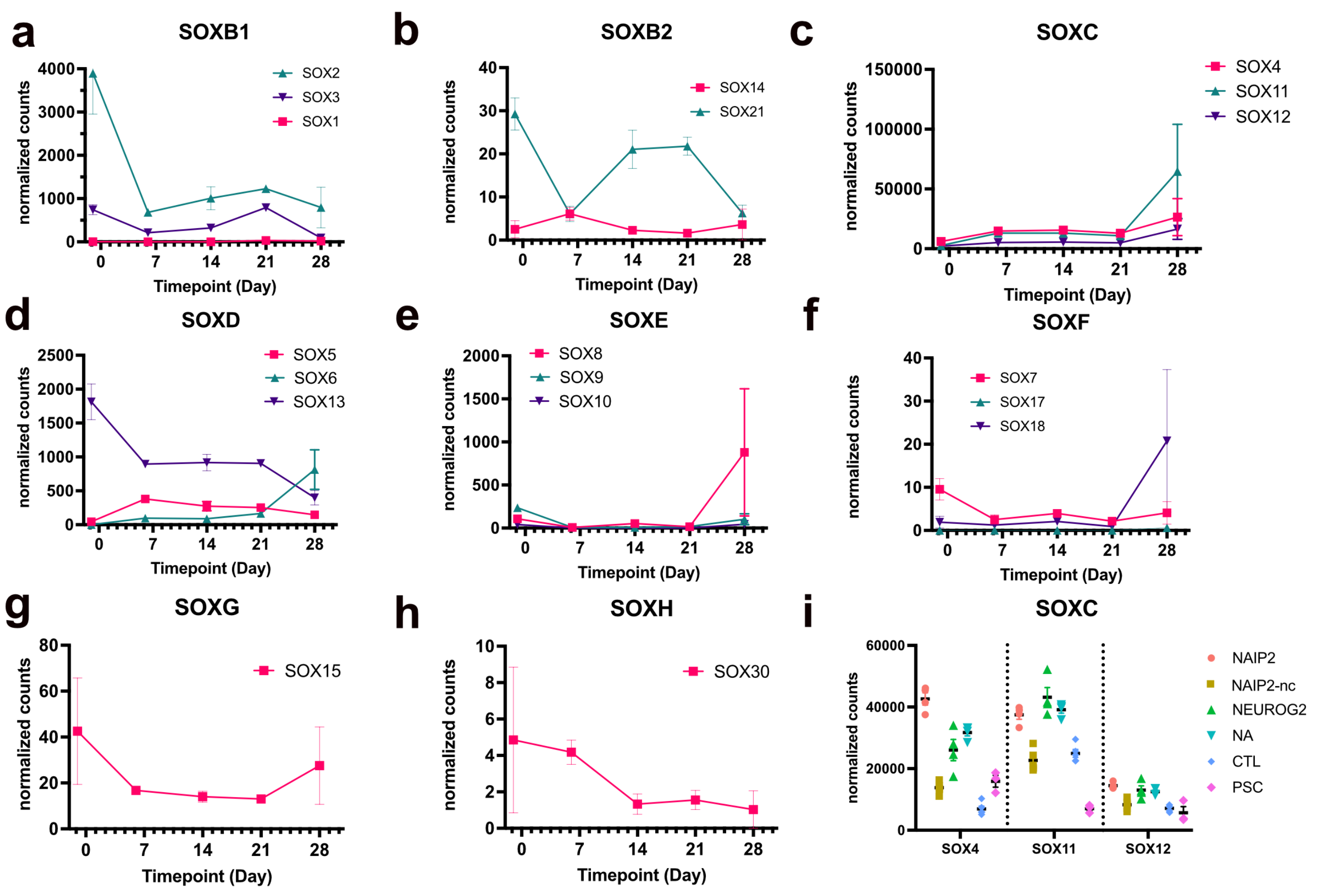

Supplementary Figure 7. ***Comparison of SOX family gene expression in RGC-iNs.*** (**a-h**) Bulk RNA-seq normalized counts for IMR90.4 NAIP2 iPSCs at 0, 1, 2, 3 and 4 weeks (n=3 for PSCs, n=4 for all other samples, error bars=SEM). (**i**) Bulk RNA-seq normalized counts compared between IMR90.4 NAIP2, NAIP2-nc, NEUROG2, NA, CTL samples differentiated for one week or undifferentiated PSCs. NAIP2-nc indicates non-clonally selected NAIP2 PSCs.

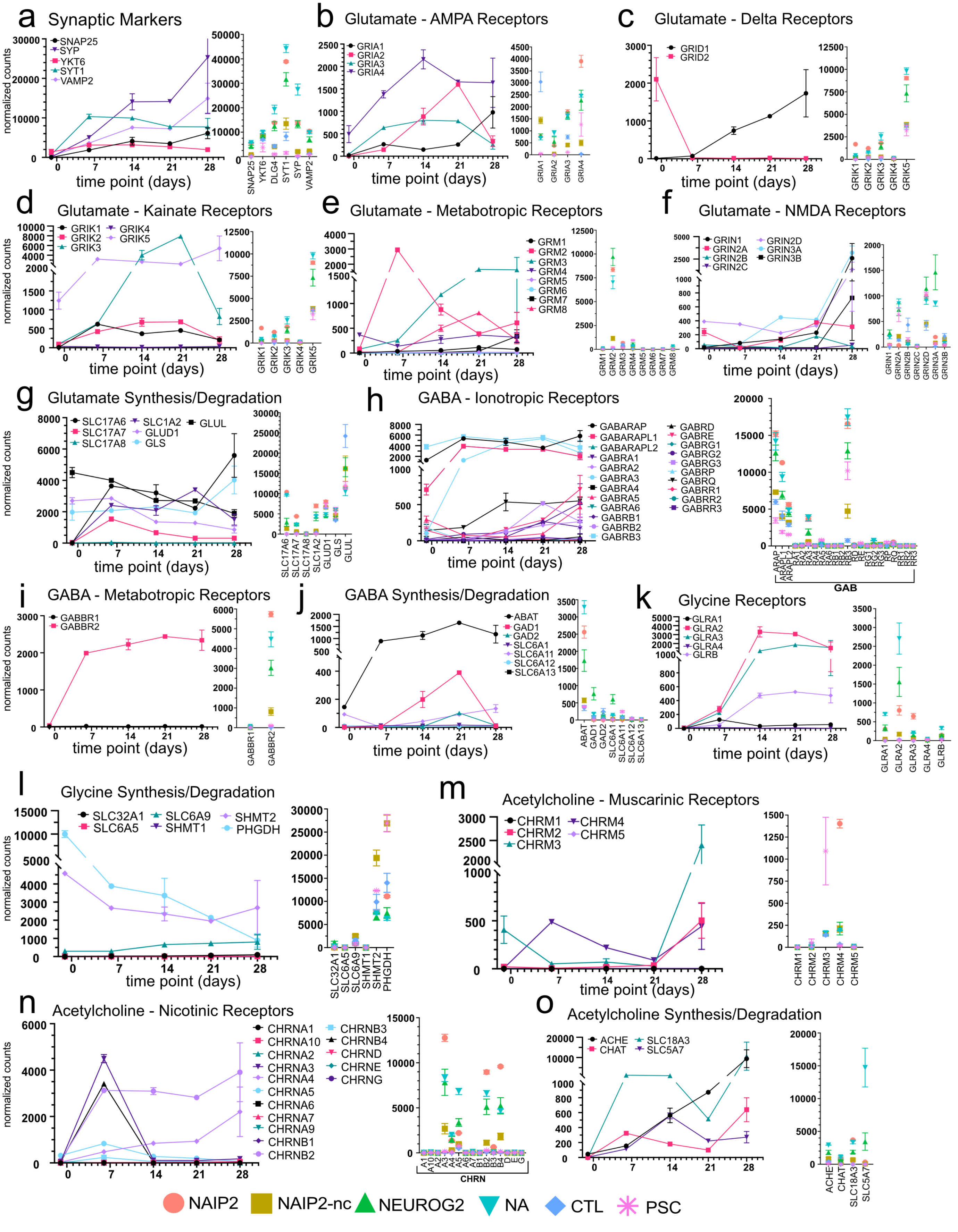
a
Synaptic Markers
SNAP25
SYP
YKT6
SYT1
VAMP2
normalized counts
time point (days)
SNAP25
YKT6
DLG4
SYT1
SYP
VAMP2
b
Glutamate - AMPA Receptors
GRIA1
GRIA2
GRIA3
GRIA4
c
Glutamate - Delta Receptors
GRID1
GRID2
GRIK1
GRIK2
GRIK3
GRIK4
GRIK5
d
Glutamate - Kainate Receptors
e
Glutamate - Metabotropic Receptors
GRM1
GRM2
GRM3
GRM4
GRM5
GRM6
GRM7
GRM8
f
Glutamate - NMDA Receptors
GRIN1
GRIN2A
GRIN2B
GRIN2C
GRIN2D
GRIN3A
GRIN3B
g
Glutamate Synthesis/Degradation
SLC17A6
SLC17A7
SLC17A8
SLC1A2
GLUD1
GLS
GLUL
h
GABA - Ionotropic Receptors
GABARAP
GABARAPL1
GABARAPL2
GABRA1
GABRA2
GABRA3
GABRA4
GABRA5
GABRA6
GABRB1
GABRB2
GABRB3
GABRD
GABRE
GABRG1
GABRG2
GABRG3
GABRP
GABRQ
GABRR1
GABRR2
GABRR3
GAB
i
GABA - Metabotropic Receptors
GABBR1
GABBR2
j
GABA Synthesis/Degradation
ABAT
GAD1
GAD2
SLC6A1
SLC6A11
SLC6A12
SLC6A13
k
Glycine Receptors
GLRA1
GLRA2
GLRA3
GLRA4
GLRB
l
Glycine Synthesis/Degradation
SLC32A1
SLC6A5
SLC6A9
SHMT1
SHMT2
PHGDH
m
Acetylcholine - Muscarinic Receptors
CHRM1
CHRM2
CHRM3
CHRM4
CHRM5
n
Acetylcholine - Nicotinic Receptors
CHRNA1
CHRNA10
CHRNA2
CHRNA3
CHRNA4
CHRNA5
CHRNA6
CHRNA7
CHRNA9
CHRNB1
CHRNB2
CHRNB3
CHRNB4
CHRND
CHRNE
CHRNG
CHRN
o
Acetylcholine Synthesis/Degradation
ACHE
CHAT
SLC18A3
SLC5A7
NAIP2
NAIP2-nc
NEUROG2
NA
CTL
PSC

Supplementary Figure 8. ***Comparison of neurotransmitter receptor and synthesis/degradation machinery in RGC-iNs over time.*** The first plot in each panel (**a-o; left**) represents bulk RNA-seq normalized counts compared between IMR90.4 NAIP2 iPSCs at 0, 1, 2, 3 and 4 weeks (n=3 for PSCs, n=4 for all other samples, error bars=SEM). The second plot in each panel (**a-o; right**) represents bulk RNA-seq normalized counts compared between IMR90.4 NAIP2, NAIP2-nc, NEUROG2, NA and CTL samples differentiated for one week or undifferentiated PSCs. NAIP2-nc indicates non-clonally selected NAIP2 PSCs. (n=3 for PSCs, n=4 for NAIP2/NEUROG2/NA, n=5 for NAIP2-nc /CTL, error bars=SEM).

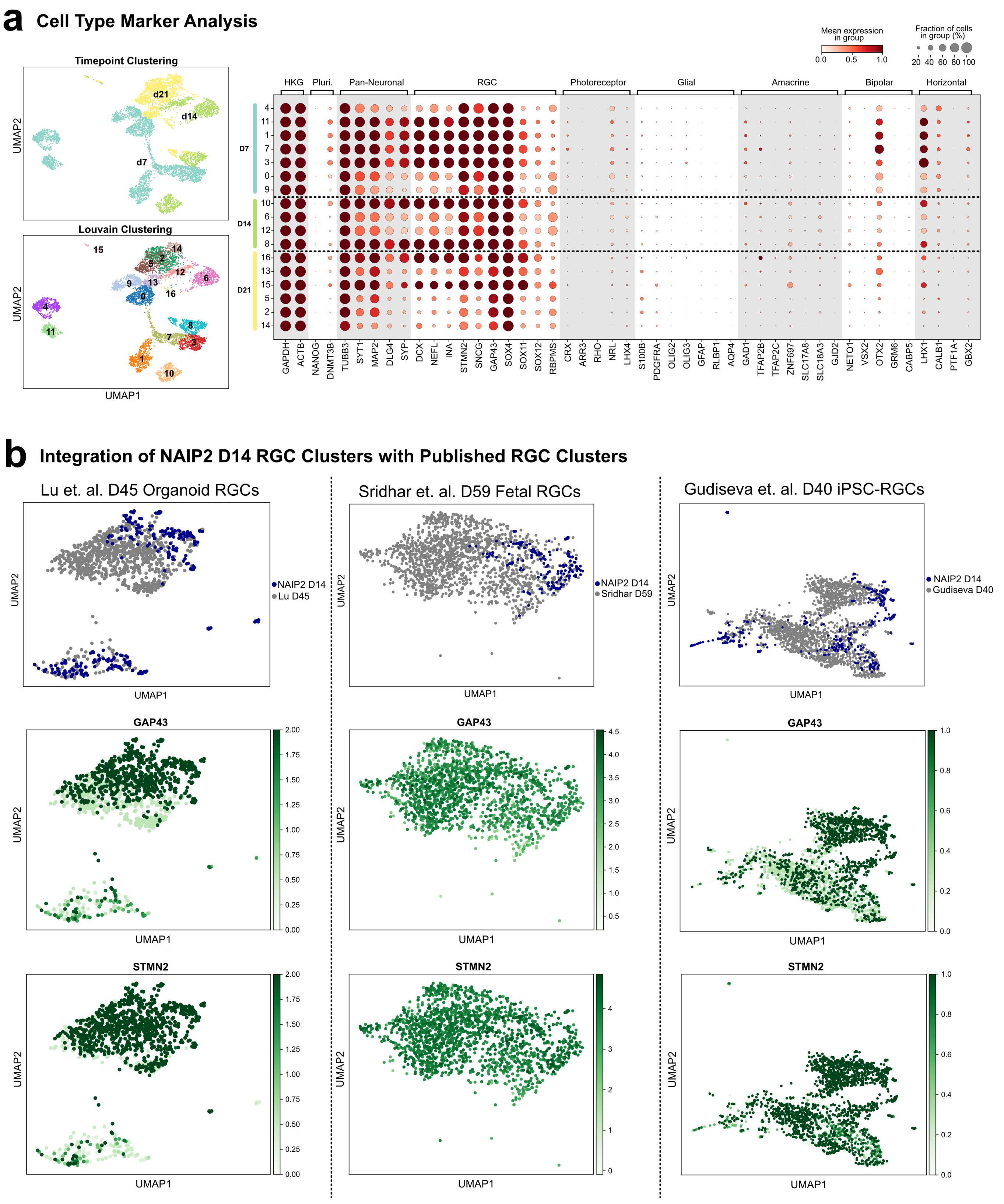

Supplementary Figure 9. ***Cell type marker and integrative data scRNA-seq analysis.*** (**a,** left) UMAPs of RGC-iNs colored by timepoint (top) and Louvain clusters (bottom). (**a,** right) Dotplot showing expression of various cell type marker genes (housekeeping [HKG], pluripotency [pluri.], pan-neuronal, RGC, photoreceptor, glial, amacrine, bipolar and horizontal cells) in each cluster of RGC-iNs across day 7, 14 and 21 timepoints. (**b**) UMAPs highlighting

the integration of NAIP2 day 14 RGC-iN clusters with published RGC clusters from day 45 retinal organoid RGCs (left), day 59 fetal RGCs (center) and day 40 iPSC-RGCs (right).

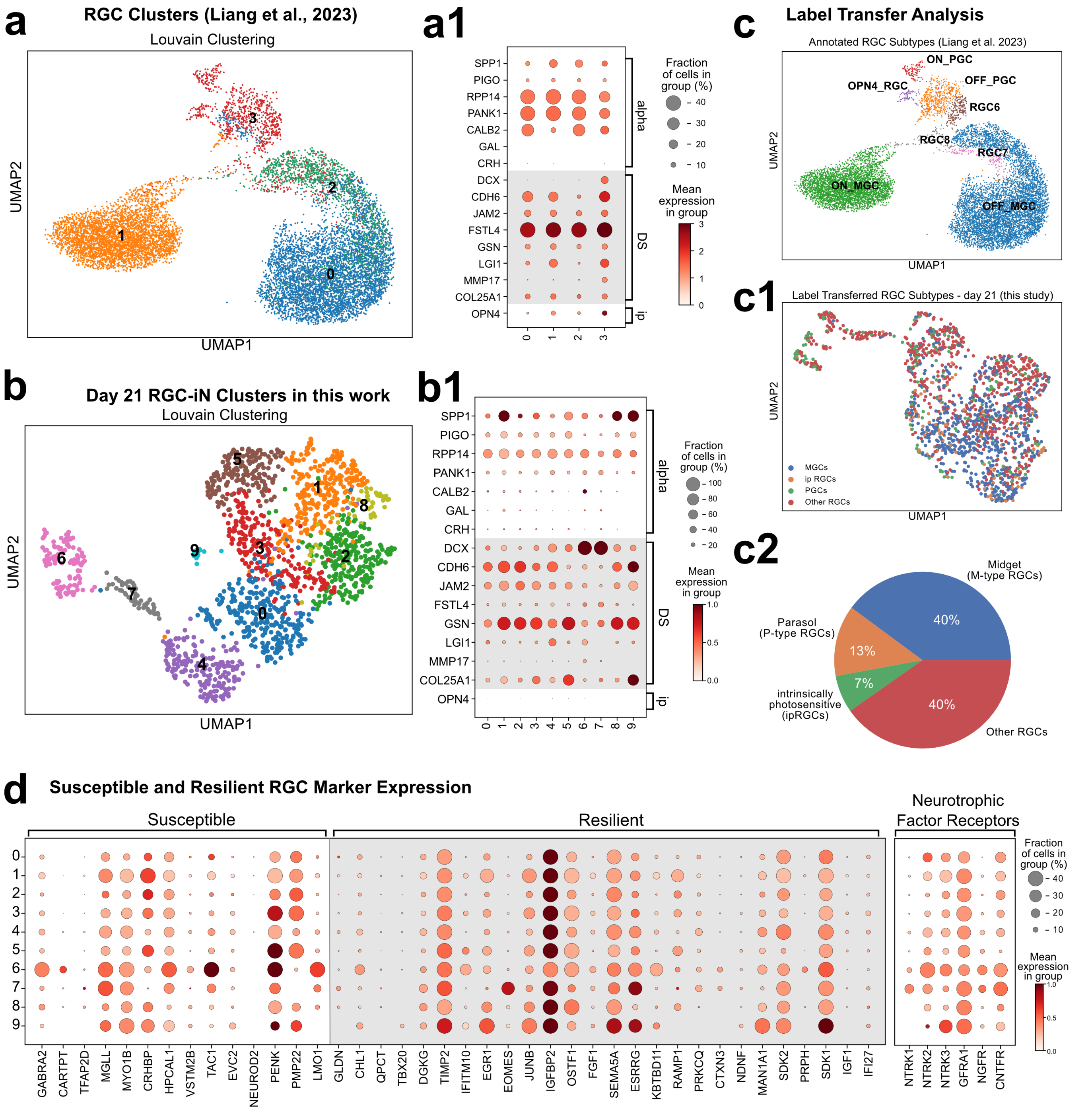

Supplementary Figure 10. ***RGC-subtypes in published scRNA-seq datasets compared with this work.*** UMAP with Louvain clustering of human RGCs from Liang et al., 2023 (**a**, left) and dotplot (**a1**, right) showing expression of various RGC-subtype marker genes in each cluster. UMAP with Louvain clustering of day 21 RGC-iNs from this work (**b**, left) and dotplot (**b1**, right) showing expression of various RGC-subtype marker genes in each cluster. (**c**) Label transfer analysis with annotated UMAP of RGC subtypes from Liang et al., 2023 (**c**, top), UMAP of label transferred RGC subtypes in day 21 RGC-iNs (**c1**, middle) and RGC-iNs subtype proportions (**c2**, bottom) in this

study. Dotplot highlighting expression of (**d**) susceptible/resilient RGC markers and neurotrophic factor receptors in day 21 RGC-iNs.

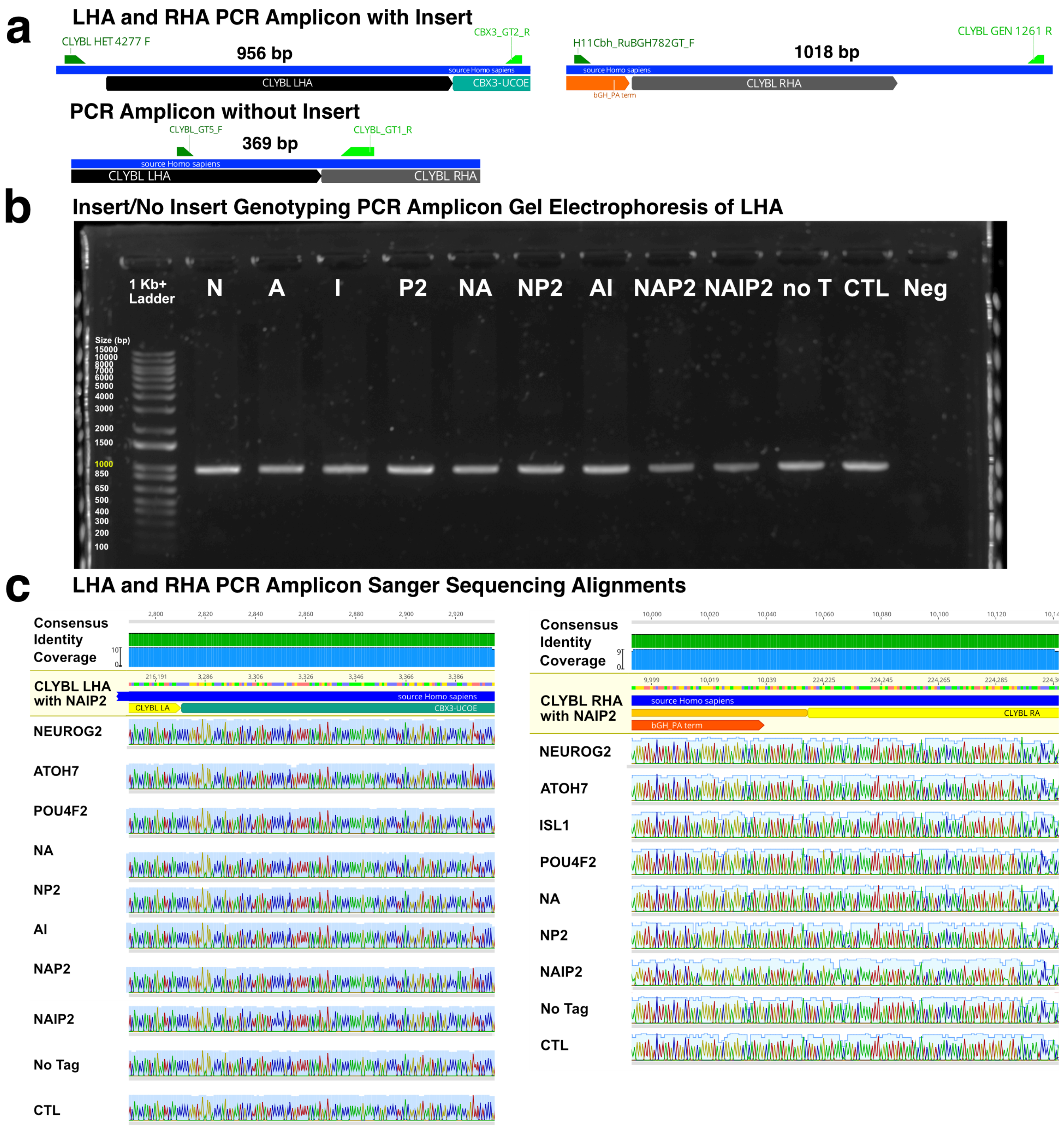

Supplementary Figure 11. ***Validation of transgene cassette insertions.*** (**a**) Diagrammatic representation of the left (LHA) and right (RHA) homology arm PCR amplicons with the transgene insert and the PCR amplicon without the insert. (**b**) Gel electrophoresis images of PCR genotyping LHA insert amplicons (956 bp) showing the presence of the transgene cassette, (**c**) Sanger sequencing verification of inserts with sequences that flank the inserts on the left and right homology arm of the CLYBL safe harbor site.

Supplementary Table 1. ***Sample characteristics and conditions.*** Table comparing the differentiation conditions of the various samples including transgene cassette, small molecule treatment, dox treatment, clonal selection and number or replicates.

Condition	CTL (control)	NEUROG2	NA	NAIP2-nc	NAIP2	PSC
**Transgene Cassette**	Empty	NEUROG2	NEUROG2-p2A-ATOH7	NEUROG2-p2A-ATOH7-p2A-ISL1-p2A-POU4F2	NEUROG2-p2A-ATOH7-p2A-ISL1-p2A-POU4F2	NEUROG2-p2A-ATOH7-p2A-ISL1-p2A-POU4F2
**Reporter**	*SIX6*-p2A-h2b-eGFP/*POU4F2*-p2A-tdTomato	*SIX6*-p2A-h2b-eGFP/*POU4F2*-p2A-tdTomato	*SIX6*-p2A-h2b-eGFP/*POU4F2*-p2A-tdTomato	*SIX6*-p2A-h2b-eGFP/*POU4F2*-p2A-tdTomato	*SIX6*-p2A-h2b-eGFP/*POU4F2*-p2A-tdTomato	*SIX6*-p2A-h2b-eGFP/*POU4F2*-p2A-tdTomato
**Small Molecule Treatment Duration**	LDN (D-1 to D0)	LDN (D-1 to D0)	LDN (D-1 to D0)	LDN (D-1 to D0)	LDN (D-1 to D0)	None
**Dox Treatment Duration**	D-1 to D6	D-1 to D6	D-1 to D6	D-1 to D6	D-1 to D6	None
**Clonal Selection Performed**	Yes	Yes	Yes	No	Yes	not applicable
**Number of Replicates**	5	4	4	4	4	3
**Timepoints Sequenced (Bulk RNAseq)**	D6, D14, D21	D6	D6	D6	D6, D14, D21, D28	D-1
**Timepoints Sequenced (scRNAseq**	None	None	None	None	D6, D14, D21	None

Supplementary Table 2. ***Key Resources Table.*** Table describing the various reagents and resources used in this work with their purpose in parentheses.

REAGENT or RESOURCE	SOURCE	IDENTIFIER
Antibodies and Imaging (for immunostaining and verification of specific protein expression)		
Calcein-AM (cell permeable dye for assessing cell viability)	Thermo Fisher Scientific	Cat# C3100MP
Chicken polyclonal anti-TAU	PhosphoSolutions	Cat# 1998-TAU, RRID: AB_2492256
Chicken polyclonal anti-MAP2	PhosphoSolutions	Cat# 1100-MAP2, RRID: AB_2492141
Click-It EdU Cell Proliferation Kit for Imaging, Alexa Fluor 647 dye (for assessing cell proliferation)	Thermo Fisher Scientific	Cat# C10340
Donkey anti-Mouse IgG (H+L) Alexa Fluor™ 488	Thermo Fisher Scientific	Cat# A-21202, RRID: AB_141607
Donkey anti-Goat IgG (H+L) Alexa Fluor™ 488	Thermo Fisher Scientific	Cat# A-11055, RRID: AB_2534102
Goat polyclonal anti-pan BRN3	Santa Cruz Biotechnology	Cat# sc-6026, RRID: AB_673441
Goat anti-Rabbit IgG (H+L) Alexa Fluor™ 488	Thermo Fisher Scientific	Cat# A-11034, RRID: AB_2576217
Goat anti-Chicken IgY (H+L) Alexa Fluor™ Plus 647	Thermo Fisher Scientific	Cat# A32933, RRID: AB_2762845
Hoechst 33342 (nuclear stain)	Thermo Fisher Scientific	Cat# H1399
Mouse monoclonal anti-ISL1	DSHB	Cat# 39.4D5, RRID: AB_2314683
Mouse monoclonal anti-PAX6	DSHB	Cat# pax6, RRID: AB_528427
Mouse monoclonal anti-MAP2	BioLegend	Cat# 801807, RRID: AB_2721423
Mouse monoclonal anti-TUJ1	Covance	Cat# MMS-435P, RRID: AB_2313773
Mouse monoclonal anti-BRN3A	Santa Cruz Biotechnology	Cat# sc-8429, RRID: AB_626765

Rabbit polyclonal anti-GLAST (SLC1A3)	Novus	Cat# NB100-1869, RRID: AB_2190597
Rabbit monoclonal anti-VIM	Abcam	Cat# ab92547, RRID: AB_10562134
Rabbit monoclonal anti-pCREB	Cell Signaling Technology	Cat# 9198, RRID: AB_2561044
Bacterial and virus strains		
NEB® Stable Competent E. coli (bacteria for plasmid growth)	New England BioLabs	Cat# C3040I
Chemicals, peptides, and recombinant proteins		
All-trans retinoic acid (ATRA) (for enhancing cell survival)	Sigma-Aldrich	Cat# R2625
Accutase (single cell passaging of hPSCs)	Sigma-Aldrich	Cat# A6964
B27 vitamin A (-) (neural supplement)	Thermo Fisher Scientific	Cat# 12587010
B27 vitamin (neural supplement)	Thermo Fisher Scientific	Cat# 17504044
BDNF (growth factor for RGC growth and survival)	Qkine	Cat# Qk050
Blebbistatin (ROCK inhibitor for improving cell survival)	Sigma-Aldrich	Cat# B0560
BrainPhys Neuronal Medium (basal media for supporting long-term growth of neurons)	StemCell Technologies	Cat# 05790
CultureOne supplement (for enhancing neural conversion)	Thermo Fisher Scientific	Cat# A3320201
DMEM (basal media)	Thermo Fisher Scientific	Cat# 11965
DMEM/F12 50:50 (basal media)	Thermo Fisher Scientific	Cat# 11330
Doxycycline hyclate (antibiotic for transgene induction)	Sigma-Aldrich	Cat# D5207
DpnI enzyme (methylation sensitive enzyme for removing parental plasmid DNA)	New England BioLabs	Cat# R0176L
F12 (basal media)	Thermo Fisher Scientific	Cat# 11765
Fetal Bovine Serum, qualified, heat inactivated (serum for maintenance of cultured cells)	Thermo Fisher Scientific	Cat# 16140071
GDNF (growth factor for enhancing RGC growth and neuronal survival)	Qkine	Cat# Qk051
GlutaMAX (stable form of glutamine as energy source for dividing cells)	Thermo Fisher Scientific	Cat# 35050061
GNE-3511 (neuroprotective DLK/LZK inhibitor)	Sigma-Aldrich	Cat# 5.33168
Insulin-Human Recombinant (N2 supplement component)	Roche	Cat# 11376497001
IWR-1-endo (WNT pathway inhibitor to promote an anterior neural fate)	EMD Millipore	Cat# 681669
L-ascorbic acid (N2 supplement component)	Sigma-Aldrich	Cat# A8960
LDN-193189 (pre-patterning BMP pathway inhibitor)	Sigma-Aldrich	Cat# SML0559
Matrigel® Growth Factor Reduced (GFR) Basement Membrane Matrix (cell attachment/differentiation of hPSCs)	Corning	Cat# 354230
mTeSR1 (maintenance and propagation of hPSCs)	Stem Cell Technologies	Cat# 85850
N-2 Supplement (neural supplement)	Thermo Fisher Scientific	Cat# 17502048
NEBuilder® HiFi DNA Assembly Mastermix (assembly of DNA fragments)	New England BioLabs	Cat# E2621S
NEAA (non-essential amino acids for supporting neuronal growth)	Thermo Fisher Scientific	Cat# 11140
Nicotinamide (NIC) (vitamin B3 supplement to enhance differentiation)	Sigma-Aldrich	Cat# 72340
Poly-L-ornithine (PLO) hydrobromide (for neural attachment)	Sigma Aldrich	Cat# P3655
Phusion Flash Polymerase (DNA amplification)	Thermo Fisher Scientific	Cat# F548L
PF-06260933 dihydrochloride (neuroprotective inhibitor of MAP4K4)	Sigma-Aldrich	Cat# PZ0272
Smoothened Agonist (SAG) (Sonic hedgehog agonist for retinal organoid differentiation)	EMD Millipore	Cat# 566660
Sodium pyruvate (carbon source supporting cellular metabolism)	Thermo Fisher Scientific	Cat# 11360
Sodium selenite (N2 supplement component)	Sigma-Aldrich	Cat# S5261
SuperScript IV reverse transcriptase (for converting mRNA into cDNA)	Thermo Fisher Scientific	Cat# 18090050

Taurine (amino acid for enhancing cell survival)	Sigma-Aldrich	Cat# T8691
Thiazovivin (ROCK inhibitor for cell survival)	LC Labs	Cat# T-9753
Transferrin - Human holo (N2 supplement component)	Sigma-Aldrich	Cat# T0665
Zeocin (antibiotic for selection of hPSCs with stably integrated gene cassette)	InvivoGen	Cat# ant-zn-1p
Critical commercial assays		
CellTiter-Glo (ATP based cell viability assay)	Promega	Cat# G7570
DNA Clean & Concentrator-5 (for PCR DNA purification)	Zymo Research	Cat# #D4013
PureLink HiPure Plasmid Midiprep Kit (for transfection grade plasmid DNA preparation)	Thermo Fisher Scientific	Cat# K210004
PureLink RNA Mini Kit (for RNA extraction)	Thermo Fisher Scientific	Cat# 12183020
Quick-RNA Miniprep Kit (for RNA extraction)	Zymo Research	Cat# R1054
ZymoPURE II Plasmid Midiprep Kit (for transfection grade plasmid DNA preparation)	Zymo Research	Cat# D4200
ZymoPURE Plasmid Miniprep Kit (for plasmid DNA preparation)	Zymo Research	Cat# D4210
Deposited data		
Raw bulk RNA-seq datasets	Sequence Read Archive	PRJNA885885
Raw single cell RNA-seq datasets	Sequence Read Archive	PRJNA973095
Experimental models: Cell lines		
Human: Passage 54 IMR90-4 iPSCs	WiCell	Cat# ips-imr90-4, RRID: CVCL_C437
Human: Passage 35 WA09 (H9) ESCs (NIH # NIHhESC-10-0062)	WiCell	Cat# WA09, RRID: CVCL_9773
Human: Passage 48 GM23720 iPSCs	Coriell	Cat# GM23720, RRID: CVCL_T818
Oligonucleotides		
H11Cbh_RuBGH782GT_F: GCATCGCATTGTCTGAGTAGGTGTCATTCTATTC	This paper	N/A
CLYBL_GEN_1261_R: TTACGGCTCTGTTGGAGAGTCCAGTATTGAATTAG	This paper	N/A
CLYBL_HET_4277_F: CATTCAGACAAGTCAGTAGGGCCATCTTAGATCATCCAGCCCTA	This paper	N/A
CBX3_GT2_R: ATTTGACTAGAAGTTGATTCGGGTGTTTCCGGAA	This paper	N/A
CLYBL_HET_2300F: CTTCTTTGTTCTTCCCCAAGTCTTTCTTTCTAGACGAAACTACTT	This paper	N/A
CLYBL_HET_2300 R: GAGAGCTCAATCCTCATCTTACATAGAAGGAAGTCAGTAGATAAT	This paper	N/A
Recombinant DNA		
pY026	Zetsche et al.[1]	RRID: Addgene_84741
AAVS1-Neo-M2rtTA	DeKelver et al.[2]	RRID: Addgene_60843
P789_pY026_EnAsCpf1_CLYBL_T1_catRNA	This paper	RRID: Addgene_202760
P931_CLYBL-CBX3-Cbh-Zeo-TetO-NAIP2	This paper	RRID: Addgene_202761
P932_CLYBL-CBX3-Cbh-Zeo-TetO-NEUROG2	This paper	RRID: Addgene_202762
Software and algorithms		
Galaxy Bioinformatics Platform	Afgan et al.[3]	https://galaxyproject.org/, RRID: SCR_006281
Python version 3.8.9	Python Software Foundation	https://www.python.org/, RRID:SCR_008394
R version 4.1.2	The R Foundation	https://www.r-project.org/, RRID:SCR_001905
ImageJ (Fiji) version 2.9.0	NIH	https://imagej.net/software/fiji/, RRID:SCR_002285
Affinity Designer version 2.1.0	Serif Ltd.	https://affinity.serif.com/, RRID:SCR_016952
GraphPad Prism 9 version 9.1.1	GraphPad Software	https://www.graphpad.com/scientific-software/prism/, RRID:SCR_002798
Geneious Prime 2022	Biomatters	https://www.geneious.com/prime/, RRID:SCR_010519

Other		
Leica DMi1 Inverted Microscope	Leica Microsystems	https://www.leica-microsystems.com/
ImageXpress Micro Confocal High-Content Imaging System	Molecular Devices	https://www.moleculardevices.com/

References for Supplementary Table 2:

1. Zetsche, B. *et al.* Multiplex gene editing by CRISPR–Cpf1 using a single crRNA array. *Nat Biotechnol* **35**, 31–34 (2017).
2. DeKelver, R. C. *et al.* Functional genomics, proteomics, and regulatory DNA analysis in isogenic settings using zinc finger nuclease-driven transgenesis into a safe harbor locus in the human genome. *Genome Res.* **20**, 1133–1142 (2010).
3. Afgan, E. *et al.* The Galaxy platform for accessible, reproducible and collaborative biomedical analyses: 2018 update. *Nucleic Acids Research* **46**, W537–W544 (2018).

Supplementary Table 3. ***SRA Accession Numbers***. Table of RNA-seq sample SRA accession numbers, names, age, cell line, replicate details and cell types.

SRA Accession	Sample Name	Age	Cell Line	Replicate	Cell Type
SAMN31111428	PSC_1	0 days	IMR-90.4	PSC biological replicate 1	Pluripotent stem cells
SAMN31111429	PSC_2	0 days	IMR-90.4	PSC biological replicate 2	Pluripotent stem cells
SAMN31111430	PSC_3	0 days	IMR-90.4	PSC biological replicate 3	Pluripotent stem cells
SAMN31111431	CTL_1	7 days	IMR-90.4	Control biological replicate 1	Control neural cells
SAMN31111432	CTL_2	7 days	IMR-90.4	Control biological replicate 2	Control neural cells
SAMN31111433	CTL_3	7 days	IMR-90.4	Control biological replicate 3	Control neural cells
SAMN31111434	CTL_4	7 days	IMR-90.4	Control biological replicate 4	Control neural cells
SAMN31111435	CTL_5	7 days	IMR-90.4	Control biological replicate 5	Control neural cells
SAMN31111436	NAIB_1	7 days	IMR-90.4	NAIB D6 biological replicate 1	Unpurified D6 retinal ganglion cells
SAMN31111437	NAIB_2	7 days	IMR-90.4	NAIB D6 biological replicate 2	Unpurified D6 retinal ganglion cells
SAMN31111438	NAIB_3	7 days	IMR-90.4	NAIB D6 biological replicate 3	Unpurified D6 retinal ganglion cells
SAMN31111439	NAIB_4	7 days	IMR-90.4	NAIB D6 biological replicate 4	Unpurified D6 retinal ganglion cells
SAMN31111440	NAIB_5	7 days	IMR-90.4	NAIB D6 biological replicate 5	Unpurified D6 retinal ganglion cells
SAMN31111441	NEUROG2_1	7 days	IMR-90.4	NEUROG2 D6 biological replicate 1	D6 Neural cells
SAMN31111442	NEUROG2_2	7 days	IMR-90.4	NEUROG2 D6 biological replicate 2	D6 Neural cells
SAMN31111443	NEUROG2_3	7 days	IMR-90.4	NEUROG2 D6 biological replicate 3	D6 Neural cells
SAMN31111444	NEUROG2_4	7 days	IMR-90.4	NEUROG2 D6 biological replicate 4	D6 Neural cells
SAMN31111445	NEUROG2_5	7 days	IMR-90.4	NEUROG2 D6 biological replicate 5	D6 Neural cells
SAMN31111446	NA_1	7 days	IMR-90.4	NA D6 biological replicate 1	D6 Neural cells
SAMN31111447	NA_2	7 days	IMR-90.4	NA D6 biological replicate 2	D6 Neural cells
SAMN31111448	NA_3	7 days	IMR-90.4	NA D6 biological replicate 3	D6 Neural cells
SAMN31111449	NA_4	7 days	IMR-90.4	NA D6 biological replicate 4	D6 Neural cells

SAMN31111450	NAIB_C1_D6_1	7 days	IMR-90.4	NAIB C1 D6 biological replicate 1	Purified D6 retinal ganglion cells
SAMN31111451	NAIB_C1_D6_2	7 days	IMR-90.4	NAIB C1 D6 biological replicate 2	Purified D6 retinal ganglion cells
SAMN31111452	NAIB_C1_D6_3	7 days	IMR-90.4	NAIB C1 D6 biological replicate 3	Purified D6 retinal ganglion cells
SAMN31111453	NAIB_C1_D6_4	7 days	IMR-90.4	NAIB C1 D6 biological replicate 4	Purified D6 retinal ganglion cells
SAMN31111454	NAIB_C1_D14_1	14 days	IMR-90.4	NAIB C1 D14 biological replicate 1	Purified D14 retinal ganglion cells
SAMN31111455	NAIB_C1_D14_2	14 days	IMR-90.4	NAIB C1 D14 biological replicate 2	Purified D14 retinal ganglion cells
SAMN31111456	NAIB_C1_D14_3	14 days	IMR-90.4	NAIB C1 D14 biological replicate 3	Purified D14 retinal ganglion cells
SAMN31111457	NAIB_C1_D14_4	14 days	IMR-90.4	NAIB C1 D14 biological replicate 4	Purified D14 retinal ganglion cells
SAMN31111458	NAIB_C1_D21_1	21 days	IMR-90.4	NAIB C1 D21 biological replicate 1	Purified D21 retinal ganglion cells
SAMN31111459	NAIB_C1_D21_2	21 days	IMR-90.4	NAIB C1 D21 biological replicate 2	Purified D21 retinal ganglion cells
SAMN31111460	NAIB_C1_D21_3	21 days	IMR-90.4	NAIB C1 D21 biological replicate 3	Purified D21 retinal ganglion cells
SAMN31111461	NAIB_C1_D21_4	21 days	IMR-90.4	NAIB C1 D21 biological replicate 4	Purified D21 retinal ganglion cells
SAMN35158738	NAIP2-D28-1	28 days	IMR-90.4	NAIP2 D28 biological replicate 1	Purified D28 retinal ganglion cells
SAMN35158739	NAIP2-D28-2	28 days	IMR-90.4	NAIP2 D28 biological replicate 2	Purified D28 retinal ganglion cells
SAMN35158740	NAIP2-D28-3	28 days	IMR-90.4	NAIP2 D28 biological replicate 3	Purified D28 retinal ganglion cells
SAMN35158741	NAIP2-D28-4	28 days	IMR-90.4	NAIP2 D28 biological replicate 4	Purified D28 retinal ganglion cells
SAMN35119890	NAIP2-D7-1	7 days	GM23720	NAIP2 D7 biological replicate 1	Purified D7 retinal ganglion cells
SAMN35119891	NAIP2-D7-2	7 days	GM23720	NAIP2 D7 biological replicate 2	Purified D7 retinal ganglion cells
SAMN35119892	NAIP2-D7-3	7 days	GM23720	NAIP2 D7 biological replicate 3	Purified D7 retinal ganglion cells
SAMN35119893	NAIP2-D14-1	14 days	GM23720	NAIP2 D14 biological replicate 1	Purified D14 retinal ganglion cells
SAMN35119894	NAIP2-D14-2	14 days	GM23720	NAIP2 D14 biological replicate 2	Purified D14 retinal ganglion cells
SAMN35119895	NAIP2-D21-1	21 days	GM23720	NAIP2 D21 biological replicate 1	Purified D21 retinal ganglion cells
SAMN35119896	NAIP2-D21-2	21 days	GM23720	NAIP2 D21 biological replicate 2	Purified D21 retinal ganglion cells
SAMN35119897	NAIP2-D21-3	21 days	GM23720	NAIP2 D21 biological replicate 3	Purified D21 retinal ganglion cells
