# Supplementary material for: Human retinal ganglion cell neurons generated by synchronous BMP inhibition and transcription factor mediated reprogramming
Source: NPJ Regen Med. 2023 Sep 29;8:55. doi: 10.1038/s41536-023-00327-x (PMC10541876; doi:10.1038/s41536-023-00327-x)
Supplement: Supplementary file 2 — reporting summary [file 41536_2023_327_MOESM2_ESM.pdf]

nature portfolio

Corresponding author(s): Wahlin, KJ

Last updated by author(s): Jun 7, 2023

Reporting Summary

Nature Portfolio wishes to improve the reproducibility of the work that we publish. This form provides structure for consistency and transparency in reporting. For further information on Nature Portfolio policies, see our Editorial Policies and the Editorial Policy Checklist.

Statistics

For all statistical analyses, confirm that the following items are present in the figure legend, table legend, main text, or Methods section.

n/a	Confirmed	
☐	☒	The exact sample size (*n*) for each experimental group/condition, given as a discrete number and unit of measurement
☐	☒	A statement on whether measurements were taken from distinct samples or whether the same sample was measured repeatedly
☐	☒	The statistical test(s) used AND whether they are one- or two-sided<br>*Only common tests should be described solely by name; describe more complex techniques in the Methods section.*
☐	☒	A description of all covariates tested
☐	☒	A description of any assumptions or corrections, such as tests of normality and adjustment for multiple comparisons
☐	☒	A full description of the statistical parameters including central tendency (e.g. means) or other basic estimates (e.g. regression coefficient) AND variation (e.g. standard deviation) or associated estimates of uncertainty (e.g. confidence intervals)
☐	☒	For null hypothesis testing, the test statistic (e.g. *F*, *t*, *r*) with confidence intervals, effect sizes, degrees of freedom and *P* value noted<br>*Give P values as exact values whenever suitable.*
☒	☐	For Bayesian analysis, information on the choice of priors and Markov chain Monte Carlo settings
☒	☐	For hierarchical and complex designs, identification of the appropriate level for tests and full reporting of outcomes
☒	☐	Estimates of effect sizes (e.g. Cohen's *d*, Pearson's *r*), indicating how they were calculated

Our web collection on statistics for biologists contains articles on many of the points above.

Software and code

Policy information about availability of computer code

Data collection	Images were acquired with an ImageXpress Micro Confocal High-Content Imaging System using MetaXpress software package, pseudocolored and merged in ImageJ (NIH). Adjustments in brightness and contrast were made using ImageJ and/or Affinity Designer (Serif Ltd.). For experiments involving direct comparisons, exposure settings were kept constant. Results were quantified using Prism 9 (v9.1.1, GraphPad software).
Data analysis	Processed single-cell feature barcode count matrices are available on GitHub (https://github.com/WahlinLab/Human_RGC-iN). For reproducibility, detailed Jupyter notebook files containing all Python code and R scripts are available on GitHub.

For manuscripts utilizing custom algorithms or software that are central to the research but not yet described in published literature, software must be made available to editors and reviewers. We strongly encourage code deposition in a community repository (e.g. GitHub). See the Nature Portfolio guidelines for submitting code & software for further information.

Data

Policy information about availability of data

All manuscripts must include a data availability statement. This statement should provide the following information, where applicable:

- Accession codes, unique identifiers, or web links for publicly available datasets
- A description of any restrictions on data availability
- For clinical datasets or third party data, please ensure that the statement adheres to our policy

Bulk and single cell RNA sequencing datasets are available as raw FASTQ files accessible at the Sequence Read Archive (SRA#; PRJNA885885, PRJNA973095). Processed single-cell feature barcode count matrices are available on GitHub (https://github.com/WahlinLab/Human_RGC-iN). For reproducibility, detailed Jupyter notebook files containing all Python code and R scripts are available on GitHub. Details about sample accession numbers, names, age, cell line, replicate and cell types can be found in Table S3.

Research involving human participants, their data, or biological material

Policy information about studies with human participants or human data. See also policy information about sex, gender (identity/presentation), and sexual orientation and race, ethnicity and racism.

Reporting on sex and gender	N/A
Reporting on race, ethnicity, or other socially relevant groupings	N/A
Population characteristics	N/A
Recruitment	N/A
Ethics oversight	N/A

Note that full information on the approval of the study protocol must also be provided in the manuscript.

Field-specific reporting

Please select the one below that is the best fit for your research. If you are not sure, read the appropriate sections before making your selection.

☒ Life sciences ☐ Behavioural & social sciences ☐ Ecological, evolutionary & environmental sciences

For a reference copy of the document with all sections, see nature.com/documents/nr-reporting-summary-flat.pdf

Life sciences study design

All studies must disclose on these points even when the disclosure is negative.

Sample size	For bulk RNA-seq experiments samples were collected in at least three independent biological triplicates. For microscopy and image analysis, images from all wells of at least three biological replicates were combined and averaged for quantification.
Data exclusions	No data was excluded from the analyses.
Replication	At least three biological replicates were used for each time point or condition in RNA-seq and image analysis experiments. An exception was day 14 single cell data for which there were only two replicates due to a failed library preparation.
Randomization	Randomization is not relevant to this study as our samples in various assays were treated uniformly and the same data analysis procedure was applied to all samples of the same experiment.
Blinding	Experiments were not blinded in this study because all results presented are based on quantitative analysis which is therefore not subject to human biases.

Reporting for specific materials, systems and methods

We require information from authors about some types of materials, experimental systems and methods used in many studies. Here, indicate whether each material, system or method listed is relevant to your study. If you are not sure if a list item applies to your research, read the appropriate section before selecting a response.

Materials & experimental systems

n/a	Involved in the study
☐	☒ Antibodies
☐	☒ Eukaryotic cell lines
☒	☐ Palaeontology and archaeology
☒	☐ Animals and other organisms
☒	☐ Clinical data
☒	☐ Dual use research of concern
☒	☐ Plants

Methods

n/a	Involved in the study
☒	☐ ChIP-seq
☒	☐ Flow cytometry
☒	☐ MRI-based neuroimaging

Antibodies

Antibodies used

Chicken polyclonal anti-TAU, PhosphoSolutions Cat# 1998-TAU, RRID: AB_2492256
Chicken polyclonal anti-MAP2, PhosphoSolutions Cat# 1100-MAP2, RRID: AB_2492141
Click-It EdU Cell Proliferation Kit for Imaging, Alexa Fluor 647 dye (for assessing cell proliferation), Thermo Fisher Scientific Cat# C10340
Donkey anti-Mouse IgG (H+L) Alexa Fluor™ 488, Thermo Fisher Scientific Cat# A-21202, RRID: AB_141607
Donkey anti-Goat IgG (H+L) Alexa Fluor™ 488, Thermo Fisher Scientific Cat# A-11055, RRID: AB_2534102
Goat polyclonal anti-pan BRN3, Santa Cruz Biotechnology Cat# sc-6026, RRID: AB_673441
Goat anti-Rabbit IgG (H+L) Alexa Fluor™ 488, Thermo Fisher Scientific Cat# A-11034, RRID: AB_2576217
Goat anti-Chicken IgY (H+L) Alexa Fluor™ Plus 647, Thermo Fisher Scientific Cat# A32933, RRID: AB_2762845
Hoechst 33342 (nuclear stain), Thermo Fisher Scientific Cat# H1399
Mouse monoclonal anti-ISL1, DSHB Cat# 39.4D5, RRID: AB_2314683
Mouse monoclonal anti-PAX6, DSHB Cat# pax6, RRID: AB_528427
Mouse monoclonal anti-MAP2, BioLegend Cat# 801807, RRID: AB_2721423
Mouse monoclonal anti-TUJ1, Covance Cat# MMS-435P, RRID: AB_2313773
Mouse monoclonal anti-BRN3A, Santa Cruz Biotechnology Cat# sc-8429, RRID: AB_626765
Rabbit polyclonal anti-GLAST (SLC1A3), Novus Cat# NB100-1869, RRID: AB_2190597
Rabbit monoclonal anti-VIM, Abcam Cat# ab92547, RRID: AB_10562134
Rabbit monoclonal anti-pCREB, Cell Signaling Technology Cat# 9198, RRID: AB_2561044

Validation

Chicken polyclonal anti-TAU, Validated by the manufacturer and multiple publications. e.g., Zhang, K.Y., et al. 2020. Role of the Internal Limiting Membrane in Structural Engraftment and Topographic Spacing of Transplanted Human Stem Cell-Derived Retinal Ganglion Cells. Stem Cell Reports,16(1),149-167.

Chicken polyclonal anti-MAP2, Validated by the manufacturer and multiple publications. e.g., Khamsing, D., et al. 2021. A role for BDNF-and NMDAR-induced lysosomal recruitment of mTORC1 in the regulation of neuronal mTORC1 activity. Molecular Brain, 14(1), pp.1-20. Allen, M., et al. 2016. Protease induced plasticity: matrix metalloproteinase-1 promotes neurostructural changes through activation of protease activated receptor 1. Scientific Reports, 6.

Click-It EdU Cell Proliferation Kit for Imaging, Alexa Fluor 647 dye (for assessing cell proliferation), Validated by the manufacturer and multiple publications. e.g., Veres TZ, et al 2009. Dendritic cell-nerve clusters are sites of T cell proliferation in allergic airway inflammation. Am J Pathol., 174(3):808-817. Hesselson D, et al. 2009. Distinct populations of quiescent and proliferative pancreatic beta-cells identified by HOTcre mediated labeling. Proc Natl Acad Sci U S A, 106(35):14896-14901.
Donkey anti-Mouse IgG (H+L) Alexa Fluor™ 488, Validated by the manufacturer and multiple publications. e.g., Puertollano R, et al. 2003. Morphology and dynamics of clathrin/GGA1-coated carriers budding from the trans-Golgi network. Mol Biol Cell, 14(4):1545-1557.

Donkey anti-Goat IgG (H+L) Alexa Fluor™ 488, Validated by the manufacturer and multiple publications. e.g., Gould E, et al. 1999. Neurogenesis in the neocortex of adult primates. Science, 286(5439):548-552.

Goat polyclonal anti-pan BRN3, Validated by the manufacturer and multiple publications. e.g., Poché RA, Furuta Y, Chaboissier MC, Schedl A, Behringer RR. Sox9 is expressed in mouse multipotent retinal progenitor cells and functions in Müller glial cell development. J Comp Neurol. 2008;510(3):237-250. Wang Q, Marcucci F, Cerullo I, Mason C. Ipsilateral and Contralateral Retinal Ganglion Cells Express Distinct Genes during Decussation at the Optic Chiasm. eNeuro. 2016;3(6):ENEURO.0169-16.2016.

Goat anti-Rabbit IgG (H+L) Alexa Fluor™ 488, Validated by the manufacturer and multiple publications. e.g., Steyn FJ, Lee K, Fogarty MJ, et al. Growth hormone secretion is correlated with neuromuscular innervation rather than motor neuron number in early-symptomatic male amyotrophic lateral sclerosis mice. Endocrinology. 2013;154(12):4695-4706. Bartel DL, Rela L, Hsieh L, Greer CA. Dendrodendritic synapses in the mouse olfactory bulb external plexiform layer. J Comp Neurol. 2015;523(8):1145-1161.

Goat anti-Chicken IgY (H+L) Alexa Fluor™ Plus 647, Validated by the manufacturer and multiple publications. e.g., Wall CE, Rose CM, Adrian M, Zeng YJ, Kirkpatrick DS, Bingol B. PPEF2 Opposes PINK1-Mediated Mitochondrial Quality Control by Dephosphorylating Ubiquitin. Cell Rep. 2019;29(10):3280-3292.e7.

Hoechst 33342 (nuclear stain), Validated by the manufacturer and multiple publications. e.g., Willard MD, Lajiness ME, Wulur IH, et al. Somatic mutations in CCK2R alter receptor activity that promote oncogenic phenotypes. Mol Cancer Res. 2012;10(6):739-749.
Mouse monoclonal anti-ISL1, Validated by the manufacturer and multiple publications. e.g., Yamamoto Y, Henderson CE. Patterns of programmed cell death in populations of developing spinal motoneurons in chicken, mouse, and rat. Dev Biol. 1999;214(1):60-71. Garcès A, Haase G, Airaksinen MS, Livet J, Filippi P, deLapeyrière O. GFRalpha 1 is required for development of distinct

subpopulations of motoneuron. J Neurosci. 2000;20(13):4992-5000.
Mouse monoclonal anti-PAX6, Validated by the manufacturer and multiple publications. e.g., Hussain MA, Miller CP, Habener JF. Brn-4 transcription factor expression targeted to the early developing mouse pancreas induces ectopic glucagon gene expression in insulin-producing beta cells. J Biol Chem. 2002;277(18):16028-16032. Lim Y, Golden JA. Expression pattern of cLhx2b, cZic1 and cZic3 in the developing chick diencephalon. Mech Dev. 2002;115(1-2):147-150.
Mouse monoclonal anti-MAP2, Validated by the manufacturer and multiple publications. e.g., Shelton MA, et al. Loss of Microtubule-Associated Protein 2 Immunoreactivity Linked to Dendritic Spine Loss in Schizophrenia. Biol Psychiatry. 2015 Sep 15;78(6):374-85. Wang X, Blanchard J, Grundke-Iqbal I, Iqbal K. Memantine Attenuates Alzheimer's Disease-Like Pathology and Cognitive Impairment. PLoS One. 2015 Dec 23;10(12):e0145441.
Mouse monoclonal anti-TUJ1, Validated by the manufacturer and multiple publications. e.g., Wang TW, Stromberg GP, Whitney JT, Brower NW, Klymkowsky MW, Parent JM. Sox3 expression identifies neural progenitors in persistent neonatal and adult mouse forebrain germinative zones. J Comp Neurol. 2006;497(1):88-100. Navarro-Quiroga I, Hernandez-Valdes M, Lin SL, Naegele JR. Postnatal cellular contributions of the hippocampus subventricular zone to the dentate gyrus, corpus callosum, fimbria, and cerebral cortex. J Comp Neurol. 2006;497(5):833-845.
Mouse monoclonal anti-BRN3A, Validated by the manufacturer and multiple publications. e.g., Nasonkin IO, Lazo K, Hambright D, Brooks M, Fariss R, Swaroop A. Distinct nuclear localization patterns of DNA methyltransferases in developing and mature mammalian retina. J Comp Neurol. 2011;519(10):1914-1930. Serrano-Saiz E, Leyva-Díaz E, De La Cruz E, Hobert O. BRN3-type POU Homeobox Genes Maintain the Identity of Mature Postmitotic Neurons in Nematodes and Mice. Curr Biol. 2018;28(17):2813-2823.e2.
Rabbit polyclonal anti-GLAST (SLC1A3), Validated by the manufacturer and multiple publications. e.g., Weng Q, Wang J, Wang J, et al. Single-Cell Transcriptomics Uncovers Glial Progenitor Diversity and Cell Fate Determinants during Development and Gliomagenesis. Cell Stem Cell. 2019;24(5):707-723.e8. Schmidt S, Luecken MD, Trümbach D, et al. Primary cilia and SHH signaling impairments in human and mouse models of Parkinson's disease. Nat Commun. 2022;13(1):4819.
Rabbit monoclonal anti-VIM, Validated by the manufacturer and multiple publications. e.g., Latil M, Nassar D, Beck B, et al. Cell-Type-Specific Chromatin States Differentially Prime Squamous Cell Carcinoma Tumor-Initiating Cells for Epithelial to Mesenchymal Transition. Cell Stem Cell. 2017;20(2):191-204.e5. Dalmas E, Lehmann FM, Dror E, et al. Interleukin-33-Activated Islet-Resident Innate Lymphoid Cells Promote Insulin Secretion through Myeloid Cell Retinoic Acid Production. Immunity. 2017;47(5):928-942.e7.
Rabbit monoclonal anti-pCREB, Validated by the manufacturer and multiple publications. e.g., Dimitrov E, Usdin TB. Tuberoinfundibular peptide of 39 residues modulates the mouse hypothalamic-pituitary-adrenal axis via paraventricular glutamatergic neurons. J Comp Neurol. 2010;518(21):4375-4394. McTague J, Ferguson M, Chik CL, Ho AK. Sustained adrenergic stimulation is required for the nuclear retention of TORC1 in male rat pinealocytes. Endocrinology. 2013;154(9):3240-3250.

Eukaryotic cell lines

Policy information about cell lines and Sex and Gender in Research

Cell line source(s)	Human: Passage 54 IMR90-4 iPSCs, WiCell Cat# ips-imr90-4, RRID: CVCL_C437<br>Human: Passage 35 WA09 (H9) ESCs (NIH # NIHhESC-10-0062), WiCell Cat# WA09, RRID: CVCL_9773<br>Human: Passage 48 GM23720 iPSCs, Coriell Cat# GM23720, RRID: CVCL_T818
Authentication	Chromosomal integrity was evaluated by copy number variation (CNV) analysis using an Infinium HumanCore-24 v1.1 BeadChip (Illumina, San Diego, CA, USA). All reporter hPSC lines were validated by differentiating them into retinal organoids.
Mycoplasma contamination	All hPSC lines were routinely tested for mycoplasma via PCR (Drexler HG, Uphoff CC. Mycoplasma contamination of cell cultures: Incidence, sources, effects, detection, elimination, prevention. Cytotechnology. 2002;39(2):75-90.).
Commonly misidentified lines<br>(See ICLAC register)	*Name any commonly misidentified cell lines used in the study and provide a rationale for their use.*
